# B-DNA Structure and Stability as Function of Nucleic Acid Composition: Dispersion-Corrected DFT Study of Dinucleoside Monophosphate Single and Double Strands

**DOI:** 10.1002/open.201300019

**Published:** 2013-08-16

**Authors:** Giampaolo Barone, Célia Fonseca Guerra, F Matthias Bickelhaupt

**Affiliations:** [a] Dipartimento di Scienze e Tecnologie Biologiche, Chimiche e Farmaceutiche, Università di PalermoViale delle Scienze, Edificio 17, 90128, Palermo (Italy) E-mail: giampaolo.barone@unipa.it; [b] Department of Theoretical Chemistry and Amsterdam Center for Multiscale Modeling, VU UniversityDe Boelelaan 1083, 1081 HV Amsterdam (The Netherlands) E-mail: f.m.bickelhaupt@vu.nl; [c] Institute for Molecules and Materials, Radboud University NijmegenHeyendaalseweg 135, 6525 AJ Nijmegen (The Netherlands)

**Keywords:** density functional calculations, DNA structures, hydrogen bonds, stacking interactions, Watson–Crick base pairs

## Abstract

We have computationally investigated the structure and stability of all 16 combinations of two out of the four natural DNA bases A, T, G and C in a di-2′-deoxyribonucleoside-monophosphate model DNA strand as well as in 10 double-strand model complexes thereof, using dispersion-corrected density functional theory (DFT-D). Optimized geometries with B-DNA conformation were obtained through the inclusion of implicit water solvent and, in the DNA models, of sodium counterions, to neutralize the negative charge of the phosphate groups. The results obtained allowed us to compare the relative stability of isomeric single and double strands. Moreover, the energy of the Watson–Crick pairing of complementary single strands to form double-helical structures was calculated. The latter furnished the following increasing stability trend of the double-helix formation energy: d(TpA)_2_ <d(CpA)_2_ <d(ApT)_2_ <d(ApA)_2_ <d(GpT)_2_ <d(GpA)_2_ <d(ApG)_2_ <d(CpG)_2_ <d(GpG)_2_ <d(GpC)_2_, where the energy differences between the last four dimers, d(ApG)_2_, d(CpG)_2_, d(GpG)_2_ and d(GpC)_2_, is within 4.0 kcal mol^−1^, and the energy between the most and the least stable isomers is 13.4 kcal mol^−1^. This trend shows that the formation energy essentially increases with the number of hydrogen bonds per base pair, that is two between A and T and three between G and C. Superimposed on this main trend are more subtle effects that depend on the order in which bases occur within a strand from the 5’- to the 3’-end.

## Introduction

The DNA double helix results from several types of stabilizing but also destabilizing interactions between and within its two complementary, intertwined polydeoxyribonucleotide chains. In fact, the major contributions to the stability of the DNA structure come from:[1] (1) hydrogen bonds in adenine–thymine (AT) and guanine–cytosine (GC) Watson–Crick pairs; (2) π–π stacking interactions among Watson–Crick paired nitrogen bases stacked along the double helix axis; (3) electrostatic repulsive interactions among the phosphate groups; (4) electrostatic attractive interactions between the phosphate groups and cations dissolved in water solution; (5) hydrophilic interactions of the sugar-phosphate skeleton with water; (6) hydrophobic interactions of the DNA cylindrical core, made up by the hydrogen-bonded and stacked nitrogen bases, with the water solvent. Recently, there has been increasing effort in developing and applying quantum chemical methods able to reproduce the structure of native B-DNA and to correctly describe the energy involved in the intrastrand and interstrand noncovalent interactions between the nucleotide monomers. This topic has been approached by both wave function methods and density functional theory.[2]

Water solvent and sodium counterions also play an important role in the formation and relative stabilization of the double-helical DNA structure because they mitigate the aforementioned long-range electrostatic repulsions among the phosphate groups in the DNA backbone and amplify π–π stacking interactions through the hydrophobic effect. This issue has been recently considered in the choice of model systems in density functional theory (DFT) calculations of the structure of dinucleoside monophosphate single strands.[3] The results obtained show that, not unexpectedly, the presence of the Na^+^ counterions at each phosphate group is even more important than the presence of the implicit solvent for providing a structure in which the relative base–base orientation and the backbone torsion angles are in better agreement with experimental B-DNA crystal structures. This result reconfirms the importance of charge neutralization in DNA model systems.

A complicating aspect of DNA in computational studies is the combination of the large size of model systems in combination with the high demand on accuracy for describing the various delicate, yet crucial types of interactions, such as, hydrogen bonding and π–π stacking. The latter can be adequately described using DFT or post-Hartree–Fock ab initio methods that properly account for electron correlation. However, the size already of minimal model systems for double-stranded DNA is prohibitive for post-Hartree–Fock approaches. For example, coupled-cluster theory with single and double electron excitations and triple electron excitations treated perturbatively, that is, CCSD(T), at the estimated complete basis set (CBS) limit, can be applied to molecules containing no more than about 50 atoms.[2b,c] On the other hand, DFT methods are able to deal with, and accurately describe these large DNA models. Hydrogen bonding in Watson–Crick base pairs and other DNA structures is well described using the generalized gradient approximation (GGA).[4] Interactions, such as π–π stacking, in which London dispersion features prominently, require the use of semilocal hybrid functionals that recover medium-range correlation effects as proposed, for example, by Truhlar and coworkers,[5] but they can also be treated very efficiently using Grimme’s empirically dispersion-corrected density functional theory (DFT-D).[6]

Model systems in pioneering DFT studies so far cover single-stranded GC dinucleoside monophosphate models (including sodium counter ions and implicit water solvent),[3] the ten possible combinations of DNA bases A, T, G and C in stacks of two Watson–Crick pairs (but without sugar-phosphate bridge between the Watson–Crick pairs),[7] and double-stranded DNA dimers (but with the sugar-phosphate bridge in the MM part of a QM/MM approach).[8]

In the present work, we extend the above quest to a quantum chemical exploration of the 16 possible single-stranded and 10 double-stranded B-DNA models that exist for the dinucleoside monophosphates of A, T, G and C, using dispersion-corrected DFT at BLYP-D/TZ2P as implemented in the Amsterdam Density Functional (ADF) program (see below). The latter approach has been shown to accurately reproduce CCSD(T) structural and energy data of stacked as well as hydrogen-bonded AT and GC complexes.4f Our DFT-D study also covers the sodium counterions at the phosphate groups as well as the effect of aqueous solvation using the conductor-like screening model (COSMO). The purpose of our work is to obtain a more detailed understanding of the structure and stability of B-DNA and how this depends on DNA-base sequences. The resulting insight is also relevant, among other things, for modeling and rationalizing the interaction of drugs with DNA, following any type of covalent[9] and/or noncovalent drug–DNA binding with particular emphasis on DNA intercalators.[10]

## Computational Details

All calculations were performed using the Amsterdam Density Functional (ADF) program developed by Baerends, Ziegler, and others,[11, 12] and the QUantum-regions Interconnected by Local Descriptions (QUILD) program by Swart and Bickelhaupt.[13] The QUILD program is a wrapper around ADF (and other programs) and is used for its superior geometry optimizer which is based on adapted delocalized coordinates.[13a] The numerical integration was performed using the procedure developed by te Velde et al.[12a,b]

Molecular orbitals (MOs) were expanded in a large uncontracted set of Slater-type orbitals (STOs) containing diffuse functions: TZ2P (no Gaussian functions are involved).[12a,b] The basis set is of triple-*ζ* quality for all atoms and has been augmented with two sets of polarization functions, that is, 3d and 4f on C, N, O and 2p, 3d on H. The 1s core shells of carbon, nitrogen and oxygen were treated by the frozen-core approximation. An auxiliary set of s, p, d, f and g STOs was used to fit the molecular density and to represent the Coulomb and exchange potentials accurately in each self-consistent field cycle.

Geometries and energies were computed with dispersion-corrected density functional theory (DFT-D) using the BLYP-D functional in which the regular BLYP functional[14, 15] is augmented with an empirical correction for long-range dispersion effects, described by a sum of damped interatomic potentials of the form C_6_R^−6^ added to the usual DFT energy.[6b, 16] The basis set superposition error (BSSE) on the bond energy is effectively absorbed into the empirical dispersion-correction potential.[16a] The starting B-DNA structures of the single and double strands were built using the NUCLEIC routine of the TINKER molecular design program package.[17]

Solvent effects in water have been estimated using the conductor-like screening model (COSMO), as implemented in the ADF program[18] with the following settings: solvent radius and dielectric constant for water were 1.9 Å and 78.4, respectively, and atomic radii were taken from the MM3 van der Waals radii scaled by 0.8333 (for details, see Table S1 of ref. [19]). The surface charges at the GEPOL93 solvent-excluding surface were corrected for outlying charges. According to the work by Riley et al.,[20] the dispersion correction does not need to be modified for the solvated systems.

The Cartesian coordinates and energies of stationary points are given in the Supporting Information.

## Results and Discussion

In the following four sections, we focus on two important findings of our investigations. The first one concerns the reliability of DFT-D in combination with the conductor-like screening model (COSMO) for mimicking bulk solvation in aqueous solution, for the calculation of dinucleoside monophosphate single and double strands in physiological conditions. The calculated structures are in fact in good agreement with experimental structures of B-DNA oligodeoxynucleotides obtained by X-ray crystallography.[21] The second important finding emerges from the analyses of the relative stability of the various model B-DNA double strands in terms of their formation energy from the corresponding single-stranded moieties as well as the energy contributions into which the formation energy can be decomposed. These findings shed light, among others, on how the overall stability and structure of B-DNA depends on its composition in terms of AT versus GC Watson–Crick pairs and the role of intramolecular interactions in genetic evolution.

There are 16 different doublet sequences and this number increases to 64 for triplet sequences in single-stranded DNA. Both combinations decrease to 10 and to 32 for doublet and triplet sequences, respectively, in double-helical DNA. In this respect, it is worth recalling that each of the DNA triplet sequences are the smallest units of genetic code in protein synthesis.[22] In the present work, we have focused our attention on DNA dimers, and we plan to extend our investigation on the structure and stability of DNA triplets in a forthcoming paper, using the same computational approach reported herein.

### Optimized structures of single- and double- stranded B-DNA dimers

**Figure 1:**
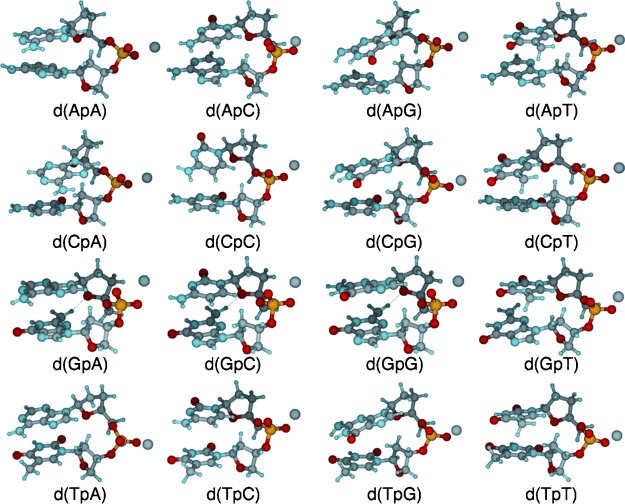
Structures of dinucleoside phosphate single strands Na-d(ApA), Na-d(ApC), Na-d(ApG), Na-d(ApT), Na-d(CpA), Na-d(CpC), Na-d(CpG), Na-d(CpT), Na-d(GpA), Na-d(GpC), Na-d(GpG), Na-d(GpT), Na-d(TpA), Na-d(TpC), Na-d(TpG) and Na-d(TpT), optimized at COSMO(H_2_O)-BLYP-D/TZ2P.

**Figure 2:**
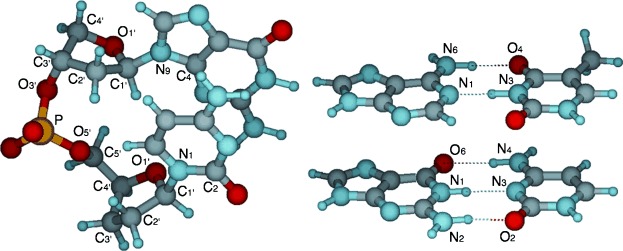
Definition of atom labels used in Tables 1–3, for d(GpC), AT and GC.

**Figure 3:**
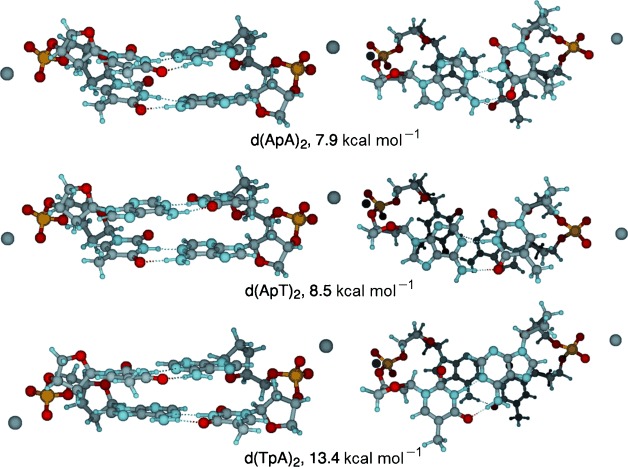
Structures and relative complexation energies Δ*E*_double,rel_ (Equation 1 b) of dinucleoside phosphate duplexes Na_2_-d(ApA)_2_, Na_2_-d(ApT)_2_ and Na_2_-d(TpA)_2_, optimized at COSMO(H_2_O)-BLYP-D/TZ2P (left: major-groove side; right: top view).

**Figure 4:**
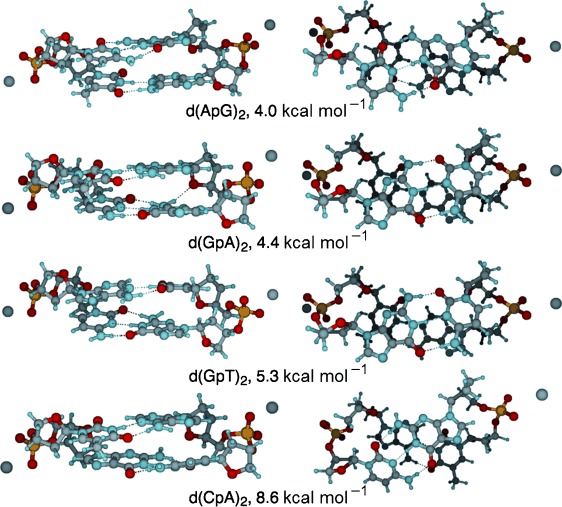
Structures and relative complexation energies Δ*E*_double,rel_ (Equation 1 b) of dinucleoside phosphate duplexes Na_2_-d(ApG)_2_, Na_2_-d(GpA)_2_, Na_2_-d(GpT)_2_ and Na_2_-d(CpA)_2_ optimized at COSMO(H_2_O)-BLYP-D/TZ2P (left: major-groove side; right: top view).

**Figure 5:**
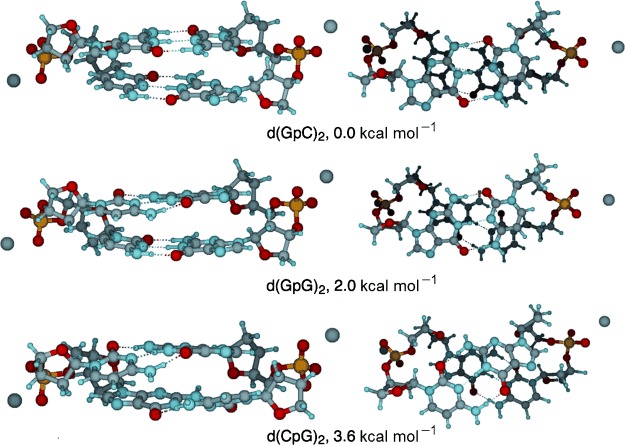
Structures and relative complexation energies Δ*E*_double,rel_ (Equation 1 b) of dinucleoside phosphate duplexes Na_2_-d(GpC)_2_, Na_2_-d(GpG)_2_ and Na_2_-d(CpG)_2_, optimized at COSMO(H_2_O)-BLYP-D/TZ2P (left: major-groove side; right: top view).

The structures of the 16 possible combinations of the four DNA nitrogen bases A, C, G and T used to build dinucleoside phosphate single strands are reported in Figure 1. The combinations of the four nitrogen bases, giving rise to 10 possible DNA doublet sequences, obtained by Watson–Crick pairing of the complementary single strands above, are reported in Figure 3–5. The geometries of both single and double strands have been fully optimized within the implicit solvent, and their corresponding Cartesian coordinates and absolute energy values are reported in Table S1 in the Supporting Information. The backbone torsion angles of the optimized structures are reported in Tables 1 and 2 and compared with available experimental crystal structures of B-DNA oligonucleotides.[21] The latter derive from a statistical analysis of 34 B-DNA oligodeoxynucleotide structures, characterized with resolutions between 0.7 and 3.3 Å. The hydrogen bond distances between Watson–Crick complementary bases are reported in Table 3. The atom labels used for defining the backbone torsion angles and the hydrogen bonding distances are shown in Figure 2.

There is satisfactory agreement between the average values of the torsion angles of both single and double strands with available experimental crystal structures of oligodeoxynucleotides in B-DNA conformation.[21] The torsion angles and hydrogen bond distances data reported in Tables 1 and 2 show that larger distortions from the B-DNA conformations, with a significantly lower value of the *β* torsion angle, have been observed for d(GpA), d(GpC) and d(GpG) in single strands and for d(GpA), d(CpA) and d(CpG) in double strands.

**Table 1:** Backbone torsion angles [°] of the 16 dinucleoside monophosphate B-DNA single strands^[a]^

ss	PO3′–C3′– C4′ (*ε*)	O5′–PO3′– C3′ (*ζ*)	C5′–O5′– PO3′ (*α*)	C4′–C5′– O5′P (*β*)	C3′–C4′– C5′–O5’ (*γ*)	C2–N1/C4–N9– C1′–O4’ (*χ*)^[b]^
d(ApT)	160.6	−84.5	−61.3	−176.2	51.0	−101.6 (6)
d(ApA)	−177.1	−78.0	−61.0	168.9	50.1	−98.8 (3)
d(TpT)	−174.4	−77.0	−64.6	167.5	50.6	−112.6 (3)
d(TpA)	−175.3	−78.2	−62.8	168.5	46.7	−95.8 (6)
d(GpT)	154.0	−80.7	−60.1	174.1	48.3	−101.6 (6)
d(ApC)	158.1	−83.8	−62.5	−170.2	50.2	−104.9 (8)
d(ApG)	−158.5	−70.4	−64.8	174.7	54.6	−114.0 (32)
d(CpT)	−174.0	−75.0	−65.1	171.5	50.3	−112.0 (1)
d(GpA)	159.7	−85.0	−81.1	−127.7	42.9	−102.5 (15)
d(TpC)	169.4	−90.7	−68.6	−161.8	40.0	−109.9 (0)
d(CpA)	−163.9	−68.8	−62.5	175.1	53.9	−102.8 (21)
d(TpG)	−162.1	−72.3	−64.0	175.5	53.1	−106.5 (29)
d(GpC)	166.2	−85.3	−79.5	−134.0	42.2	−116.9 (10)
d(CpG)	−163.0	−71.6	−62.5	175.9	53.3	−104.6 (27)
d(GpG)	160.3	−82.2	−82.3	−123.6	47.9	−100.6 (25)
d(CpC)	168.0	−84.1	−79.4	−141.7	44.7	−111.7 (7)
Av (SD)^[c]^	176.8 (16)	−79.2 (6)	−67.6 (8)	−170.2 (24)	48.8 (4)	−106.1 (17)
Exptl (SD)^[d]^	184 (11)	−95 (10)	−62 (15)	176 (9)	48 (11)	−102 (14)

[a] Computed at BLYP-D/TZ2P and COSMO for simulating aqueous solution. See Figure 2 for definitions and Figure 1 for structures. [b] Average of the two *χ* angles present in each dinucleoside monophosphate (see Figure 2). [c] Average with standard deviation (SD) over the 16 dinucleoside monophosphate single strands. [d] Experimental values with standard deviation (SD) from X-ray crystal structures of oligodeoxynucleotides in B-DNA conformation (ref. [21]).

**Table 2:** Backbone torsion angles [°] of the 10 dinucleoside monophosphate B-DNA double strands^[a]^

ds	ss^[b]^	PO3′–C3′– C4’ (*ε*)	O5′–PO3′– C3’ (*ζ*)	C5′–O5′– PO3’ (*α*)	C4′–C5′– O5′P (*β*)	C3′–C4′– C5′–O5’ (*γ*)	C2–N1/C4–N9– C1′–O4’ (*χ*)^[c]^
d(ApT)_2_	d(ApT)	154.5	−81.8	−60.9	−173.3	50.9	−97.8 (6)
	d(ApT)	153.6	−81.3	−60.9	−172.6	50.6	−97.7 (6)
d(ApA)_2_	d(ApA)	158.0	−82.4	−63.1	−171.8	48.4	−93.8 (5)
	d(TpT)	164.5	−86.8	−62.9	−174.2	50.7	−105.3 (2)
d(TpA)_2_	d(TpA)	164.1	−90.1	−69.6	−152.7	45.4	−106.2 (3)
	d(TpA)	164.9	−90.2	−70.4	−152.5	42.2	−106.0 (4)
d(GpT)_2_	d(GpT)	148.4	−79.4	−59.7	−169.2	51.8	−95.4 (6)
	d(ApC)	148.9	−79.8	−59.1	−170.4	51.8	−97.0 (6)
d(ApG)_2_	d(ApG)	150.9	−79.2	−62.2	−167.7	48.4	−99.7 (7)
	d(CpT)	164.6	−87.4	−64.8	−164.6	49.3	−107.0 (4)
d(GpA)_2_	d(GpA)	158.2	−84.7	−78.5	−131.5	43.5	−102.9 (11)
	d(TpC)	169.9	−92.2	−67.6	−162.0	48.3	−112.4 (1)
d(CpA)_2_	d(CpA)	164.0	−85.9	−78.5	−132.8	41.0	−101.0 (8)
	d(TpG)	164.8	−90.1	−70.6	−149.7	44.2	−106.5 (3)
d(GpC)_2_	d(GpC)	158.6	−86.4	−65.6	−159.9	48.3	−106.8 (4)
	d(GpC)	158.9	86.0	−67.2	−156.8	47.8	−107.6 (3)
d(CpG)_2_	d(CpG)	164.4	−86.7	−76.7	−136.3	41.2	−103.0 (5)
	d(CpG)	164.1	−86.2	−77.1	−135.8	41.1	−103.0 (5)
d(GpG)_2_	d(GpG)	152.0	−82.6	−63.7	−159.4	47.5	−99.0 (4)
	d(CpC)	170.3	−90.8	−69.3	−160.0	46.8	−111.5 (4)
Av (SD)^[d]^		159.9 (7)	−76.9 (38)	−67.4 (6)	−157.7 (14)	47.0 (4)	−103.0 (7)
Exptl (SD)^[e]^		184 (11)	−95 (10)	−62 (15)	176 (9)	48 (11)	−102 (14)

[a] Computed at BLYP-D/TZ2P and COSMO for simulating aqueous solution. See Figure 2 for definitions and Figure 3–5 for structures. [b] Single strands that constitute the double strand. [c] Average of the two *χ* angles present in each dinucleoside monophosphate (see Figure 2). [d] Average with standard deviation (SD) over the 10 dinucleoside monophosphate double strands. [e] Experimental values with standard deviation (SD) from X-ray crystal structures of oligodeoxynucleotides in B-DNA conformation (ref. [21]).

The hydrogen bond lengths obtained are also in good agreement with those calculated with the same DFT-D method and basis set for isolated DNA nitrogen bases.[4g] However, the presence of the sugar phosphate chain induces small distortions in the hydrogen-bonded base pairs. These distortions are somewhat more pronounced in the case of AT than GC base pairs. For example, there is a closer match between the distances of isolated AT base pairs and those in d(TpA), whereas N6–O4 and N1–N3 distances are slightly shorter and longer, by 0.1 Å, respectively, in d(ApT) (see Table 3 and ref. [4g]). On the other hand, there is an excellent agreement, within 0.03 Å, between the three O6–N4, N1–N3 and N2–O2 distances of isolated GC base-pairs and those in d(GpC)_2_, d(CpG)_2_ and d(GpG)_2_ (see Table 3 and ref. [4g]).

**Table 3:** Hydrogen-bond structure (in Å) and stability (in kcal mol^−1^) of the 10 dinucleoside monophosphate B-DNA double strands^[a]^

ds	ss 1/ss 2^[b]^	Distance					Δ*E*_strain_^[c]^	Δ*E*_int_^[c]^	Δ*E*_double_^[c]^	Δ*E*_double,rel_^[c]^
		N6–O4 (AT)	N1–N3 (AT)	O6–N4 (GC)	N1–N_3_ (GC)	N2–O2 (GC)				
d(ApA)_2_	d(ApA)	2.88	2.81	–	–	–	2.9	−22.1	−19.2	7.9
	d(TpT)	2.99	2.77	–	–	–	–	–	–	–
d(ApT)_2_	d(ApT)	2.93	2.78	–	–	–	3.6	−22.1	−18.7	8.5
	d(ApT)	2.93	2.78	–	–	–	–	–	–	–
d(TpA)_2_	d(TpA)	2.92	2.79	–	–	–	10.3	−24.0	−13.8	13.4
	d(TpA)	2.92	2.79	–	–	–	–	–	–	–
										
d(ApG)_2_	d(ApG) d(CpT)	2.88	2.80	2.87	2.88	2.80	2.6	−25.7	−23.1	4.0
d(GpA)_2_	d(GpA) d(TpC)	2.98	2.78	2.81	2.88	2.88	3.0	−25.7	−22.7	4.4
d(GpT)_2_	d(ApC) d(GpT)	2.88	2.82	2.85	2.87	2.82	5.4	−27.2	−21.9	5.3
d(CpA)_2_	d(CpA) d(TpG)	2.92	2.78	2.84	2.86	2.84	9.4	−27.9	−18.5	8.6
d(GpC)_2_	d(GpC)	–	–	2.84	2.90	2.81	4.3	−31.4	−27.1	0.0
	d(GpC)	–	–	2.84	2.89	2.82	–	–	–	–
d(GpG)_2_	d(CpC)	–	–	2.81	2.89	2.84	4.9	−29.9	−25.1	2.0
	d(GpG)	–	–	2.84	2.88	2.84	–	–	–	–
d(CpG)_2_	d(CpG)	–	–	2.85	2.85	2.85	11.3	−34.8	−23.5	3.6
	d(CpG)	–	–	2.85	2.85	2.85	–	–	–	–
(AT)_2_	AT	2.89	2.80	–	–	–	–	–	−19.1	–
	AT	2.89	2.81	–	–	–	–	–	–	–
(GC)_2_	GC	–	–	2.84	2.88	2.84	–	–	−25.1	–
	GC	–	–	2.84	2.88	2.84	–	–	–	–
	AT^[d]^	2.91	2.82	–	–	–	–	–	−9.8	–
	GC^d^	–	–	2.85	2.90	2.84	–	–	−13.6	-

**Figure 6:**
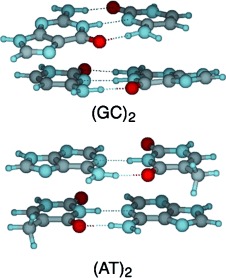
Structures of duplexes (GC)_2_ and (AT)_2_, optimized at COSMO(H_2_O)-BLYP-D/TZ2P.

[a] Computed at BLYP-D/TZ2P and COSMO for simulating aqueous solution. Parameters of the two base pair dimers (AT)_2_ and (GC)_2_ have been calculated for comparison. See Figure 2 for definitions and Figure 3–6 for structures. [b] Single strands that constitute the double strand. [c] See Equations 1 and 3. [d] From ref. [4g].

### Double-strand stability

In the following, we show how the relative stability of the double-stranded dimers emerges from an interplay between the strain energy in the single strands and the interaction between these single strands. Especially trends in the latter can be well understood in terms of molecular structural features. We analyze the stability of a DNA double helix in terms of the (relative) complexation energy associated with forming the double strand from two single strands, Δ*E*_double_ (or Δ*E*_double,rel_), see Equations 1:


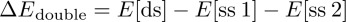
(1)


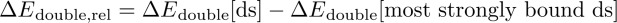
(2)

This analysis leads to a global order of stabilities among all of our DNA double strands which are collected in Table 3. The major categorization in terms of stability order can be made, not unexpectedly, in terms of the number of AT and GC Watson–Crick pairs. The stability Δ*E*_double_ of the model double helix increases as the number of GC pairs increases from zero (−14 to −19 kcal mol^−1^) to one (−19 to −23 kcal mol^−1^) to two (−24 to −27 kcal mol^−1^). Differences in energy between isomeric *single* strands are significantly smaller, that is, less than 1 kcal mol^−1^ between TpG and GpT, between CpA and ApC, between GpA and ApG, and between ApT and TpA; 1.2 kcal mol^−1^ between CpT and TpC, and 1.6 kcal mol^−1^ between CpG and GpC (see Table S1).

However, also *within* a family of isomers, consisting of the same number of AT and GC pairs, there are significant fluctuations in stability. As can be seen in Table 3, the following orders of stability in terms of complexation energy Δ*E*_double_ can be observed for the three families of model double helices (see Equations 2):


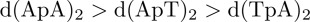
(2a)


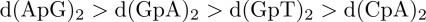
(2b)


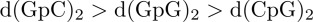
(2c)

This is in line with our earlier finding that the affinity of a model template–primer complex for an incoming new DNA base not only depends on the complexation energy of the new Watson–Crick (or mismatched) base pair but also on the terminal base in the primer strand to which the incoming DNA base binds via π–π stacking interactions.[7a]

### Analysis of double-strand interaction

To obtain a better understanding of the in silico observed trends, we have decomposed the formation energy Δ*E*_double_ of a double strand from two complementary single strands, into two main energy contributions: 1) the strain Δ*E*_strain_ that is the energy required to distort the isolated single strands from their individual equilibrium structure into the geometry they assume in the double strand; and 2) the interaction energy Δ*E*_int_ that is the difference between the energy of the double helix and that of the two single strands with the same geometry they assume in the double helix (see Equation 3):


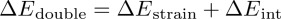
(3)

The results of our analyses for the 10 double helix models are collected in Table 3. In the first place, it appears that, for a given family of double helical isomers, the trend in relative stability Δ*E*_double_ always follows the amount of strain energy Δ*E*_strain_ associated with deforming the single strands to the geometry they have to adopt to form Watson–Crick pairs in the overall system. For example, along d(ApA)_2_, d(ApT)_2_, and d(TpA)_2_, the strain energy Δ*E*_strain_ increases from 2.9 to 3.6 to 10.3 kcal mol^−1^ and, accordingly, the complexation energy Δ*E*_double,rel_ weakens from −19.1 to −18.7 to −13.8 kcal mol^−1^ (see Table 3). This interesting trend is related to the increasing structural distortion of the complementary single strands when they form the double-stranded complex. In general, all the values of torsion angles of the free single-stranded structures, reported in Table 1, change when they form the double-stranded structures (see Table 2). Remarkably, the value of *β* of the single-stranded doublets, which involves the atoms C4′–C5′–O5′P (see Figure 2), experiences the largest modification in the less stable double-stranded structures, namely d(TpA)_2_ d(CpA)_2_ and d(CpG)_2_ (see Table 3). In more detail, the value of *β* changes by some 34° for d(TpG), 38° for d(TpA), 48° for d(CpG), and 52° for d(CpA) (compare Tables 1 and 2). Inspection of Figure 3–5 (top view) reveals that, in all four of these dinucleotide strands, the associated internal rotations around the C5′–O5’ bond leads to a less favorable π–π stacking arrangement between the two stacked DNA bases in the resulting double helices than in the separate single strands. Likewise, the more stable double-stranded isomers with the less strained single-strand fragments display more favorable π–π stacking arrangements between the stacked DNA bases within a single strand. This can be understood as a conflict, arising from the structural characteristics of the dinucleotides d(TpG), d(TpA), d(CpG) and d(CpA), between, on one hand, the requirement to adopt a proper arrangement for entering into interstrand Watson–Crick pairing and, on the other hand, an optimal intrastrand π–π stacking arrangement. The interaction energy Δ*E*_int_ behaves in a less systematic manner than Δ*E*_strain_. Along the same series, d(ApA)_2_, d(ApT)_2_, and d(TpA)_2_, Δ*E*_int_ first remains essentially constant at −22.1 and −22.1 kcal mol^−1^ and then strengthens to −24.0 kcal mol^−1^.

### Contribution of the sugar phosphate backbone

To quantify the effect of the backbone on the stability of the Watson–Crick pairs in the duplex structure, we also compare them with those in simple Watson–Crick pairs and in the simple *stacks* of Watson–Crick pairs, that is, stacks without a sugar–phosphate–sugar backbone. Previously, we found that Δ*E*_double_ in aqueous solution for simple AT and GC Watson–Crick pairs amounts to −9.8 and −13.6 kcal mol^−1^, respectively, computed at the same level of theory as used in this study.[4g] If we compare this to the present model duplexes, we see that the latter have effectively slightly weaker Watson–Crick bonds per base pair. For example, in the strongest AT bound duplex, that is, d(ApA)_2_, the effective Watson–Crick bond strength per base pair amounts to Δ*E*_double_/2=−9.6 kcal mol^−1^. Apparently, the slight increase in deformation strain Δ*E*_strain_ that occurs if a backbone is present somewhat reduces the effective stability of the Watson–Crick interaction. As this strain becomes larger, in d(TpA)_2_, the effective stability is further reduced to Δ*E*_double_/2=−6.9 kcal mol^−1^.

Thus, we have computed the complexation energy Δ*E*_double_ associated with forming simple (AT)_2_ stacks and (GC)_2_ stacks from the corresponding “single strands” of AT+AT stacks and GC+GC stacks, respectively, each of them without the sugar–phosphate–sugar backbone. The equilibrium structures of (AT)_2_ and (GC)_2_ are shown in Figure 6, wheras the corresponding hydrogen-bond distances and complexation energies Δ*E*_double_ are reported in Table 3. These backboneless systems were obtained by removing the sugar phosphate units from d(ApT)_2_ and d(GpC)_2_, substituting C1’ with a H atom, and then optimizing the structure of the stacked base pairs (AT)_2_ and (GC)_2_. Likewise, the constituting single strand analogues were obtained by removing, in addition, one of the two AT stacks from (AT)_2_ and one of the two GC stacks from (GC)_2_, followed by optimization of the remaining stack of DNA bases. The computed complexation energies Δ*E*_double_ appear to be −19.1 and −25.1 for (AT)_2_ and (GC)_2_, respectively. This corresponds to effective Watson–Crick bond strengths per base pair of Δ*E*_double_/2=−9.6 and −12.6 kcal mol^−1^, respectively. This is almost, that is, within a kcal mol^−1^, the same effective complexation energy as for simple base pairs.

We conclude that the geometrical constraints imposed by the backbone reduce the stability of B-DNA by 0–3 kcal mol^−1^ and 0–2 kcal mol^−1^ per AT and GC base pair, respectively. This weakening shows up in the aforementioned strain energy Δ*E*_strain_, a somewhat weakened interaction energy Δ*E*_int_, but also in slightly (i.e., by a few hundredth of an Å) stretched or compressed hydrogen-bond distances (see Table 3). As soon as the backbone is removed from the double-helical model systems, the effective Watson–Crick complexation energy is enhanced and approaches that of the isolated base pairs. Likewise, hydrogen-bond distances in (AT)_2_ and (GC)_2_ relax and essentially adopt the values in the corresponding isolated base pairs (see Table 3).4f Thus, the values for N6–O4 and N1–N3 distances with an AT base pair of (AT)_2_ are, on the average, 2.80 and 2.90 Å, respectively, whereas the values for O6–N4, N1–N3 and N2–O2 distances within a GC base pair in (GC)_2_ amount to 2.84, 2.88 and 2.84 Å, respectively.

## Conclusions

Our systematic computational analysis of the structure and the stability of all possible single strands and double strands of dinucleoside monophosphates with sodium counter ions in aqueous solution is the first quantum chemical study that copes with these large model systems using dispersion-corrected DFT and implicit solvation (COSMO). Our computations agree well with the available experimental data, notably X-ray crystallographic structures of oligodeoxynucleotides in a B-DNA conformation.

We propose a physical model for understanding trends in stability for the formation of double-stranded from single-stranded DNA dimers through a decomposition of the complexation energy into two counterbalancing contributions: the stabilizing interaction energy between two complementary single strands, and the destabilizing strain energy that is required to deform the single strands from their own equilibrium structure to the geometry they adopt in the double helix. The major contribution to the overall trend in stability of the double strand stems from Watson–Crick hydrogen bonding contained in the interaction term Δ*E*_int_ between the complementary single strands. The effective strength of the hydrogen-bonding interaction Δ*E*_int_ is somewhat less in the model double helices than in individual base pairs but it still follows the number of hydrogen bonds. Consequently, the stability Δ*E*_double_ of the model double helix increases as the number of GC pairs increases from zero (−14 to −19 kcal mol^−1^) to one (−19 to −23 kcal mol^−1^) to two (−24 to −27 kcal mol^−1^).

The reason that the hydrogen-bonding interaction Δ*E*_int_ is weaker in the model double helices is the geometrical constraint imposed by the backbone. In addition to preventing the hydrogen bonds to adopt their optimal Watson–Crick geometry, the deformation of the backbone, upon bending the single strands into the right shape for entering into Watson–Crick complexation, also contributes a strain energy term Δ*E*_strain_ that further weakens the overall complexation energy Δ*E*_double_=Δ*E*_strain_+Δ*E*_int_ for forming the model double helices. Interestingly, the stability of model double helices with a particular number of AT and GC pairs varies significantly depending on other structural features, namely: (1) the partitioning of purine and pyrimidine bases over the two single strands; and (2) the order in which two DNA bases appear in the 5′→3’ direction along a strand. This trend, which is superimposed on that of the interaction term, stems from a variation in strain energy.

## Supplementary material

As a service to our authors and readers, this journal provides supporting information supplied by the authors. Such materials are peer reviewed and may be re-organized for online delivery, but are not copy-edited or typeset. Technical support issues arising from supporting information (other than missing files) should be addressed to the authors.
