# Supplementary material for: B-DNA Structure and Stability as Function of Nucleic Acid Composition: Dispersion-Corrected DFT Study of Dinucleoside Monophosphate Single and Double Strands
Source: ChemistryOpen. 2013 Aug 16;2(5-6):186–93. doi: 10.1002/open.201300019 (PMC3892189; doi:10.1002/open.201300019)
Supplement: Supplementary file 1 [file open0002-0186-SD1.pdf]

ChemistryOPEN

Supporting Information

© 2013 The Authors. Published by Wiley-VCH Verlag GmbH & Co. KGaA, Weinheim

B-DNA Structure and Stability as Function of Nucleic Acid Composition: Dispersion-Corrected DFT Study of Dinucleoside Monophosphate Single and Double Strands

Giampaolo Barone,*[a] Célia Fonseca Guerra,[b] and F. Matthias Bickelhaupt*[b, c]

open_201300019_sm_miscellaneous_information.pdf

Content

Table S1. Cartesian coordinates and ADF total bonding energies of the following Watson-Crick base pairs, stacked Watson-Crick base pairs, single- and double-stranded di-deoxyribo-nucleoside-monophosphates, computed at COSMO(H_2O)-BLYP-D/TZ2P:

AT, $(AT)_2$, GC, $(GC)_2$, $Na_2d(ApA)_2$, $Na_2d(ApG)_2$, $Na_2d(ApT)_2$, $Na_2d(CpA)_2$, $Na_2d(CpG)_2$, $Na_2d(GpA)_2$, $Na_2d(GpC)_2$, $Na_2d(GpG)_2$, $Na_2d(GpT)_2$, $Na_2d(TpA)_2$, Nad(ApA), Nad(ApC), Nad(ApG), Nad(ApT), Nad(CpA), Nad(CpC), Nad(CpG), Nad(CpT), Nad(GpA), Nad(GpC), Nad(GpG), Nad(GpT), Nad(TpA), Nad(TpC), Nad(TpG), Nad(TpT).

Table S1. Cartesian coordinates (in Å) and ADF total bonding energies (in kcal/mol) of Watson-Crick base pairs, stacked Watson-Crick base pairs, single- and double-stranded di-deoxyribo-nucleoside-monophosphates, computed at COSMO(H_2O)-BLYP-D/TZ2P.

AT, Total Bonding Energy: -4490.00 kcal/mol

H	6.867350	-2.900038	0.922624
N	6.551788	-3.142425	-0.010236
C	6.091012	-2.266203	-0.970197
N	5.720755	-2.866772	-2.089564
C	5.953668	-4.217360	-1.841398
C	5.797091	-5.377210	-2.636208
N	5.352584	-5.324321	-3.918208
N	6.092849	-6.583238	-2.085607
C	6.552463	-6.619544	-0.815732
N	6.781292	-5.592306	0.022226
C	6.462089	-4.408990	-0.544885
H	6.054771	-1.201451	-0.785469
H	5.428399	-6.160178	-4.487445
H	5.379878	-4.432232	-4.398663
H	6.771524	-7.611255	-0.425912
H	10.192373	-4.783402	-0.701876
N	9.736970	-4.486867	-1.558634
C	9.417867	-5.481223	-2.464476
O	9.659543	-6.680354	-2.274049
N	8.799587	-4.999830	-3.609302
C	8.498488	-3.658638	-3.921202
O	7.930136	-3.391697	-4.994726
C	8.901787	-2.685865	-2.913099
C	8.630637	-1.225057	-3.159911
C	9.497368	-3.146276	-1.779738
H	8.528882	-5.700163	-4.296593
H	9.128551	-0.887676	-4.079150
H	8.990279	-0.620721	-2.319487
H	7.553492	-1.057625	-3.286880
H	9.818729	-2.486192	-0.982116

$(AT)_2$, Total Bonding Energy: -8999.14 kcal/mol

H	7.751734	-3.190982	0.966916
N	7.131197	-3.360462	0.183476
C	6.294214	-2.439110	-0.408910
N	5.582643	-2.946919	-1.403079

C	5.985012	-4.277734	-1.461496
C	5.598471	-5.355071	-2.295668
N	4.685840	-5.253705	-3.278740
N	6.184729	-6.562164	-2.065774
C	7.098157	-6.692532	-1.084140
N	7.541630	-5.748136	-0.239089
C	6.946630	-4.556307	-0.474198
H	6.253158	-1.415644	-0.062666
H	4.492685	-6.061425	-3.886875
H	4.319009	-4.341799	-3.518172
H	7.516216	-7.691358	-0.978286
H	10.778168	-4.026710	-1.074815
N	10.069129	-3.939635	-1.795259
C	9.748903	-5.092516	-2.495874
O	10.287885	-6.187946	-2.271870
N	8.783985	-4.910232	-3.465275
C	8.169442	-3.703242	-3.807984
O	7.335354	-3.673289	-4.741985
C	8.568278	-2.535477	-3.032812
C	7.932610	-1.202406	-3.332107
C	9.504533	-2.710467	-2.060560
H	8.514394	-5.776859	-4.012418
H	8.115594	-0.908368	-4.374876
H	8.333893	-0.427016	-2.669280
H	6.845581	-1.264542	-3.191368
H	9.859289	-1.896118	-1.438550
H	7.668570	-11.521899	-6.990117
N	7.359733	-10.557253	-7.031237
C	6.488906	-10.017300	-7.953457
N	6.279392	-8.722433	-7.774877
C	7.058792	-8.403601	-6.667135
C	7.282855	-7.187387	-5.976912
N	6.710295	-6.016964	-6.312494
N	8.144049	-7.218951	-4.923007
C	8.735882	-8.376575	-4.569681
N	8.598299	-9.582152	-5.143945
C	7.745749	-9.535145	-6.192970
H	6.047059	-10.626605	-8.729728
H	6.870258	-5.181966	-5.732475
H	5.994986	-6.006765	-7.027883
H	9.403181	-8.306187	-3.713288

H	6.173870	-12.007042	-3.587054
N	5.756096	-11.126170	-3.867941
C	6.113018	-10.005569	-3.133316
O	6.903146	-10.050996	-2.177134
N	5.507231	-8.840531	-3.557432
C	4.571967	-8.718808	-4.588342
O	4.066342	-7.601169	-4.840905
C	4.241499	-9.944026	-5.304733
C	3.256436	-9.886084	-6.443549
C	4.851280	-11.094054	-4.907472
H	5.767314	-7.966476	-3.018519
H	2.284818	-9.505178	-6.099799
H	3.113061	-10.881417	-6.879848
H	3.622844	-9.204402	-7.222164
H	4.661840	-12.049774	-5.383559

GC, Total Bonding Energy: -4396.70 kcal/mol

H	1.798027	-1.953196	0.764976
N	1.916480	-2.756605	0.156870
C	1.369753	-2.919761	-1.101144
N	1.697797	-4.076638	-1.652218
C	2.499646	-4.700354	-0.699282
C	3.183767	-5.955537	-0.740638
O	3.170035	-6.832495	-1.623604
N	3.973958	-6.135297	0.428625
C	4.060127	-5.260164	1.495345
N	4.837380	-5.641252	2.541651
N	3.399218	-4.106355	1.539380
C	2.652048	-3.883607	0.432268
H	0.744722	-2.159494	-1.547707
H	4.489636	-7.011801	0.473587
H	5.489110	-6.409091	2.438814
H	5.046132	-4.946957	3.249382
H	7.838652	-3.696474	0.374147
N	7.143416	-3.788126	-0.360425
C	7.377281	-4.766511	-1.347696
O	8.408965	-5.474541	-1.262847
N	6.452377	-4.900294	-2.344775
C	5.372245	-4.096273	-2.365473
N	4.475712	-4.264017	-3.369514
C	5.145425	-3.068502	-1.387837

C	6.061877	-2.954491	-0.386200
H	4.556967	-5.100635	-3.937994
H	3.538912	-3.889849	-3.247486
H	4.272677	-2.427364	-1.435833
H	5.990422	-2.231478	0.417440

$(GC)_2$, Total Bonding Energy: -8818.53 kcal/mol

H	2.191738	-1.663807	0.675994
N	2.080205	-2.496702	0.108256
C	1.370333	-2.593473	-1.074981
N	1.402484	-3.810479	-1.590340
C	2.177620	-4.545609	-0.694276
C	2.580585	-5.914113	-0.716237
O	2.254941	-6.795206	-1.549644
N	3.444988	-6.224912	0.348316
C	3.827904	-5.345308	1.345628
N	4.650294	-5.833993	2.299402
N	3.409232	-4.075943	1.406146
C	2.613555	-3.737608	0.367643
H	0.856233	-1.739910	-1.494680
H	3.835083	-7.199953	0.371861
H	5.089781	-6.765173	2.215302
H	5.026079	-5.174140	2.968930
H	7.477672	-3.385563	0.923227
N	6.894624	-3.674166	0.143701
C	7.121656	-4.952087	-0.387581
O	8.025411	-5.663939	0.121457
N	6.348298	-5.357623	-1.430117
C	5.400208	-4.533546	-1.936216
N	4.648298	-4.991516	-2.950605
C	5.192308	-3.207652	-1.424215
C	5.959899	-2.819473	-0.369599
H	4.695384	-5.985985	-3.228186
H	3.848618	-4.444664	-3.246535
H	4.431465	-2.559545	-1.844028
H	5.876557	-1.853607	0.113055
H	7.286203	-12.675483	-3.264894
N	6.682388	-11.891223	-3.485980
C	5.570303	-11.917960	-4.308220
N	4.982814	-10.738042	-4.409099
C	5.753740	-9.897569	-3.607179

C	5.611066	-8.510557	-3.304764
O	4.764099	-7.708546	-3.770116
N	6.563729	-8.076389	-2.366046
C	7.567771	-8.862987	-1.828957
N	8.402216	-8.262683	-0.952602
N	7.746044	-10.148986	-2.152426
C	6.813829	-10.604549	-3.018262
H	5.254096	-12.828201	-4.798473
H	6.489723	-7.079622	-2.042576
H	8.216257	-7.315325	-0.585155
H	9.070052	-8.851976	-0.471189
H	6.892639	-10.624927	1.892996
N	6.160750	-10.425273	1.218169
C	5.531834	-9.174744	1.306795
O	5.898885	-8.378727	2.208747
N	4.553147	-8.886422	0.407388
C	4.198516	-9.797633	-0.529492
N	3.240313	-9.454948	-1.406145
C	4.809962	-11.095742	-0.598359
C	5.801360	-11.365702	0.294094
H	2.899307	-8.480375	-1.452392
H	3.059812	-10.068736	-2.191533
H	4.512718	-11.813440	-1.354619
H	6.349789	-12.299144	0.321733

$Na_2d(ApA)_2$, Total Bonding Energy: -16803.53 kcal/mol

H	0.236453	-0.420787	-0.151360
C	1.014588	-0.219629	0.600389
O	1.685458	-1.457785	0.989272
C	2.712821	-1.737605	0.003816
C	2.122594	0.628697	-0.016507
C	2.902693	-0.435575	-0.811313
O	2.869790	1.236909	1.087003
H	0.571507	0.217799	1.498680
H	3.612092	-2.039078	0.547106
H	1.746190	1.426504	-0.662634
H	2.453557	-0.506393	-1.804085
H	3.952600	-0.184162	-0.937536
N	2.338369	-2.894133	-0.824449
C	1.637442	-2.940852	-2.020846
N	1.479647	-4.165407	-2.495449

C	2.106711	-4.977188	-1.555982
C	2.307467	-6.376723	-1.488738
N	1.868431	-7.242410	-2.420333
N	2.989840	-6.857999	-0.412674
C	3.439059	-6.004972	0.531142
N	3.299289	-4.672171	0.573428
C	2.635837	-4.211397	-0.507526
H	1.266077	-2.045288	-2.498079
H	2.069474	-8.246421	-2.327623
H	1.436592	-6.888333	-3.263436
H	3.987713	-6.470973	1.346535
P	4.411694	1.775013	0.913138
O	4.674532	2.286992	-0.491310
O	4.646884	2.738260	2.064854
O	5.304174	0.413801	1.086905
C	5.244632	-0.273981	2.376339
C	6.280172	-1.387749	2.424298
O	5.868445	-2.482295	1.537127
C	6.915486	-2.740841	0.568534
C	7.706196	-0.984173	1.972649
C	7.761326	-1.462443	0.509176
H	8.448683	-1.516997	2.576857
H	4.238266	-0.690162	2.514251
H	5.455620	0.441932	3.181283
H	6.280665	-1.772839	3.452086
H	7.508419	-3.604147	0.890771
H	7.873373	0.093055	2.068587
H	7.280802	-0.728879	-0.144187
H	8.774067	-1.668849	0.150245
N	6.301026	-3.128088	-0.694668
C	5.656699	-2.310247	-1.607481
N	5.102839	-2.967588	-2.610813
C	5.396351	-4.301918	-2.343490
C	5.077062	-5.504982	-3.016210
N	4.338363	-5.570455	-4.145053
N	5.562877	-6.659591	-2.488007
C	6.285388	-6.634269	-1.350555
N	6.613348	-5.563062	-0.613043
C	6.142690	-4.422534	-1.159626
H	5.647621	-1.238601	-1.477236
H	4.028644	-6.489333	-4.482561

H	3.828950	-4.745627	-4.437896
H	6.623333	-7.604594	-0.999285
H	7.142508	-13.908622	-5.755216
C	7.809642	-13.957193	-4.882248
O	7.969898	-12.630564	-4.288369
C	6.895697	-12.418633	-3.335117
C	7.164089	-14.797682	-3.785315
C	6.189073	-13.783584	-3.164650
O	8.229077	-15.230582	-2.878615
H	8.798891	-14.312609	-5.181996
H	7.331000	-12.059503	-2.401460
H	6.648657	-15.681946	-4.170072
H	5.249172	-13.826711	-3.718970
H	5.971956	-13.997117	-2.121082
N	5.996458	-11.337399	-3.805007
C	6.094565	-10.068518	-3.203328
O	6.896001	-9.800573	-2.302183
N	5.206347	-9.130043	-3.691846
C	4.331105	-9.272550	-4.761385
O	3.625776	-8.303299	-5.125105
C	4.328013	-10.583932	-5.397215
C	3.425673	-10.831995	-6.579582
C	5.161808	-11.534219	-4.894624
H	5.288967	-8.172459	-3.237109
H	3.660502	-10.139018	-7.399179
H	2.375056	-10.661825	-6.306534
H	3.536738	-11.860338	-6.941743
H	5.213405	-12.520232	-5.343551
P	7.893875	-15.830313	-1.384532
O	6.521654	-16.475206	-1.337356
O	9.088926	-16.687112	-1.000273
O	7.810270	-14.486911	-0.452338
C	9.026140	-13.683755	-0.323237
C	8.805385	-12.567414	0.683688
O	7.857519	-11.600634	0.123464
C	6.797854	-11.365895	1.081270
C	8.222168	-13.007738	2.053520
C	6.731422	-12.629292	1.951287
H	8.705191	-12.441963	2.857480
H	9.281241	-13.258587	-1.302497
H	9.850509	-14.320797	0.022150

H	9.771970	-12.062682	0.812792
H	7.032495	-10.489495	1.690308
H	8.370477	-14.077093	2.232105
H	6.176070	-13.422441	1.443320
H	6.266161	-12.421495	2.919637
N	5.572292	-11.031541	0.338203
C	5.112058	-9.704457	0.338401
O	5.621989	-8.801594	1.013905
N	4.019725	-9.473581	-0.475007
C	3.352126	-10.401955	-1.267949
O	2.369460	-10.046039	-1.958127
C	3.874673	-11.758436	-1.227359
C	3.194228	-12.824344	-2.047367
C	4.959532	-11.995465	-0.441983
H	3.636517	-8.486127	-0.451292
H	2.125578	-12.871240	-1.800094
H	3.643865	-13.804800	-1.865595
H	3.264910	-12.592507	-3.119156
H	5.418422	-12.975403	-0.388786
Na	7.559430	-18.619985	-0.570482
Na	5.355943	4.510313	0.450331

$Na_2d(ApG)_2$, Total Bonding Energy: -16714.19 kcal/mol

H	0.140729	-0.437780	0.743655
C	1.006275	-0.189686	1.376301
O	1.770407	-1.393833	1.693613
C	2.653738	-1.667810	0.578961
C	1.985626	0.684537	0.596011
C	2.700660	-0.372067	-0.269709
O	2.833879	1.371951	1.573504
H	0.674467	0.254137	2.318461
H	3.624102	-1.948703	0.995845
H	1.488630	1.441603	-0.017118
H	2.134144	-0.473927	-1.198134
H	3.716956	-0.088114	-0.534264
N	2.194757	-2.836609	-0.190192
C	1.313463	-2.894521	-1.260763
N	1.161609	-4.111199	-1.757200
C	1.985748	-4.905217	-0.967100
C	2.285694	-6.287880	-0.998114
N	1.762411	-7.148977	-1.890063

N	3.150609	-6.757038	-0.056612
C	3.676267	-5.911009	0.853870
N	3.464041	-4.592478	0.972833
C	2.621630	-4.140481	0.020955
H	0.810483	-2.012626	-1.632300
H	2.036259	-8.140438	-1.872286
H	1.157129	-6.799504	-2.621286
H	4.356602	-6.373617	1.565547
P	4.378032	1.832454	1.261692
O	4.564094	2.269909	-0.180360
O	4.735080	2.838912	2.343388
O	5.220682	0.440617	1.437858
C	5.211838	-0.192293	2.757773
C	6.271368	-1.282522	2.820499
O	5.856438	-2.419834	1.990897
C	6.870118	-2.687245	0.989701
C	7.673236	-0.867482	2.309148
C	7.688499	-1.394604	0.862125
H	8.445788	-1.364911	2.905791
H	4.217048	-0.619132	2.940229
H	5.433289	0.560560	3.525322
H	6.310157	-1.625474	3.862323
H	7.492940	-3.528509	1.312056
H	7.820374	0.215698	2.363066
H	7.170551	-0.695600	0.199222
H	8.693057	-1.593772	0.476326
N	6.221171	-3.116523	-0.241126
C	5.471243	-2.338191	-1.113566
N	4.971195	-3.021314	-2.123807
C	5.413595	-4.327285	-1.911766
C	5.213545	-5.520921	-2.668398
O	4.534317	-5.668759	-3.716973
N	5.896814	-6.618949	-2.112555
C	6.670065	-6.578815	-0.967574
N	7.317237	-7.713041	-0.628741
N	6.820554	-5.476577	-0.225180
C	6.190684	-4.402664	-0.744704
H	5.351858	-1.279608	-0.937403
H	5.786395	-7.541178	-2.601986
H	7.225437	-8.577591	-1.184846
H	7.697785	-7.767510	0.307175

H	7.234866	-14.350010	-5.466572
C	7.867914	-14.311907	-4.568436
O	8.033989	-12.926663	-4.125981
C	7.002934	-12.617609	-3.146898
C	7.165577	-15.019112	-3.413061
C	6.207764	-13.920641	-2.930327
O	8.184382	-15.386416	-2.427290
H	8.859403	-14.717424	-4.787873
H	7.488392	-12.280784	-2.230578
H	6.635364	-15.923167	-3.725238
H	5.308338	-13.958372	-3.549682
H	5.908256	-14.045069	-1.892253
N	6.180803	-11.476008	-3.605893
C	6.390530	-10.186575	-3.030540
O	7.305089	-10.033916	-2.192179
N	5.579837	-9.164292	-3.415359
C	4.657515	-9.331660	-4.383994
N	3.888958	-8.280627	-4.707799
C	4.509141	-10.589949	-5.062667
C	5.290525	-11.620552	-4.643472
H	4.070691	-7.351739	-4.295371
H	3.224212	-8.364094	-5.466298
H	3.804661	-10.711889	-5.877749
H	5.247145	-12.599135	-5.106509
P	7.764830	-15.821546	-0.896023
O	6.363807	-16.401802	-0.848165
O	8.901599	-16.684718	-0.376608
O	7.703795	-14.387900	-0.106567
C	8.954282	-13.640969	0.034765
C	8.801355	-12.557366	1.091128
O	7.916477	-11.505079	0.586910
C	6.815620	-11.315498	1.503470
C	8.192161	-13.026274	2.437511
C	6.712223	-12.610067	2.324810
H	8.676720	-12.496214	3.264825
H	9.210734	-13.189105	-0.932078
H	9.756343	-14.324384	0.341351
H	9.796958	-12.118492	1.237722
H	7.012467	-10.457626	2.150817
H	8.313444	-14.103966	2.584271
H	6.147389	-13.369770	1.777814

H	6.235095	-12.428154	3.292766
N	5.620704	-10.963875	0.714254
C	5.096674	-9.663631	0.802813
O	5.504559	-8.812625	1.604479
N	4.071324	-9.398289	-0.083856
C	3.460157	-10.296727	-0.953006
O	2.502614	-9.925038	-1.669713
C	4.001363	-11.647107	-0.950163
C	3.338658	-12.690363	-1.813583
C	5.071320	-11.898658	-0.147941
H	3.699418	-8.404801	-0.062027
H	2.260071	-12.713020	-1.609151
H	3.754171	-13.683012	-1.621072
H	3.455944	-12.453945	-2.879484
H	5.563761	-12.865076	-0.134068
Na	7.240126	-18.494919	0.165052
Na	5.416055	4.484716	0.586310

$Na_2d(ApT)_2$, Total Bonding Energy: -16804.94 kcal/mol

H	5.544949	-0.806823	1.591592
C	6.416574	-1.181860	2.149326
O	6.695953	-2.569774	1.789751
C	7.448042	-2.560547	0.551899
C	7.671242	-0.425430	1.720372
C	7.994251	-1.121436	0.384560
O	8.672848	-0.611605	2.772433
H	6.223963	-1.145888	3.224260
H	8.232503	-3.315385	0.639962
H	7.503466	0.647466	1.590536
H	7.475175	-0.583757	-0.410978
H	9.054911	-1.113598	0.148323
N	6.602483	-2.986586	-0.575415
C	5.713552	-2.240459	-1.335246
N	5.060098	-2.952752	-2.238350
C	5.539670	-4.247019	-2.062440
C	5.234437	-5.474406	-2.700756
N	4.325796	-5.609698	-3.683917
N	5.899397	-6.579976	-2.267884
C	6.791194	-6.481967	-1.262098
N	7.137818	-5.384209	-0.575042
C	6.486280	-4.293308	-1.027945

H	5.588012	-1.178232	-1.177968
H	4.152516	-6.533451	-4.098297
H	3.849860	-4.792960	-4.043407
H	7.282766	-7.414651	-0.996560
P	10.288862	-0.517506	2.496982
O	10.620803	0.475784	1.398959
O	10.925342	-0.296187	3.859069
O	10.652553	-2.001107	1.906666
C	10.395474	-3.149014	2.777265
C	10.941897	-4.415872	2.139486
O	10.143434	-4.740607	0.954051
C	11.018632	-4.881988	-0.192393
C	12.415005	-4.342064	1.660526
C	12.290135	-4.089924	0.144630
H	12.910279	-5.300956	1.848452
H	9.312951	-3.243608	2.931570
H	10.889964	-2.993545	3.744939
H	10.804056	-5.222068	2.871786
H	11.252388	-5.937786	-0.347439
H	12.968644	-3.548828	2.172189
H	12.151506	-3.022961	-0.046659
H	13.147225	-4.454472	-0.429941
N	10.281930	-4.449531	-1.390820
C	9.780760	-5.427307	-2.268272
O	10.010600	-6.637505	-2.149226
N	9.010733	-4.932773	-3.301340
C	8.690430	-3.603962	-3.552567
O	7.981558	-3.305705	-4.541996
C	9.231687	-2.637488	-2.608131
C	8.937924	-1.173592	-2.812986
C	9.981274	-3.110779	-1.576626
H	8.628857	-5.667089	-3.967957
H	9.263029	-0.854328	-3.812000
H	9.448409	-0.562720	-2.062309
H	7.857755	-0.982515	-2.751682
H	10.385325	-2.447548	-0.821212
H	6.977702	-11.381940	-10.209232
C	7.439393	-12.157809	-9.579733
O	8.060749	-11.558334	-8.401677
C	7.017305	-11.328569	-7.424583
C	6.359233	-13.072009	-9.006737

C	5.814428	-12.202312	-7.857371
O	7.016361	-14.311809	-8.587787
H	8.216785	-12.686284	-10.136709
H	7.420917	-11.597875	-6.446085
H	5.579839	-13.323694	-9.731555
H	4.997196	-11.595163	-8.250807
H	5.419628	-12.788629	-7.031889
N	6.684046	-9.896491	-7.344130
C	5.830831	-9.148265	-8.141974
N	5.809125	-7.861688	-7.833935
C	6.704888	-7.750462	-6.775006
C	7.145567	-6.643874	-6.007939
N	6.735275	-5.377126	-6.201992
N	8.055555	-6.893442	-5.027677
C	8.513016	-8.145756	-4.831518
N	8.186723	-9.255212	-5.509769
C	7.268547	-8.997059	-6.463736
H	5.252453	-9.603259	-8.934097
H	7.103010	-4.623109	-5.609153
H	6.031226	-5.178918	-6.900412
H	9.224562	-8.247742	-4.015793
P	6.429311	-15.283821	-7.402085
O	4.912619	-15.270347	-7.354521
O	7.112882	-16.626924	-7.597296
O	6.943373	-14.549730	-6.031289
C	8.389035	-14.414149	-5.847851
C	8.687587	-13.882045	-4.455766
O	8.237244	-12.490043	-4.365559
C	7.341721	-12.345179	-3.235343
C	7.997061	-14.635147	-3.289286
C	6.781826	-13.747194	-2.954794
H	8.678056	-14.688406	-2.432832
H	8.781011	-13.723671	-6.605692
H	8.866830	-15.395333	-5.965588
H	9.779363	-13.892794	-4.341893
H	7.898053	-11.972817	-2.372147
H	7.706941	-15.650262	-3.577356
H	5.951638	-13.981137	-3.625867
H	6.447691	-13.838823	-1.916747
N	6.353095	-11.304351	-3.560543
C	6.496577	-10.029375	-2.985637

O	7.353484	-9.753232	-2.136397
N	5.588855	-9.090804	-3.432920
C	4.579096	-9.275316	-4.369903
O	3.823564	-8.322747	-4.674008
C	4.482417	-10.613088	-4.935371
C	3.407875	-10.899738	-5.952418
C	5.378733	-11.545408	-4.514350
H	5.694396	-8.124409	-3.004217
H	2.418949	-10.655251	-5.542658
H	3.418777	-11.953446	-6.246910
H	3.545402	-10.278674	-6.848203
H	5.383436	-12.549050	-4.922328
Na	4.871761	-17.745347	-7.686530
Na	12.073223	1.750470	2.978465

$Na_2d(CpA)_2$, Total Bonding Energy: -16711.98 kcal/mol

H	0.112887	0.625648	-0.457246
C	0.748891	0.374189	0.402061
O	0.780378	-1.084505	0.576500
C	2.072864	-1.581993	0.107189
C	2.194273	0.783577	0.121498
C	2.686728	-0.424055	-0.687480
O	2.863511	0.915042	1.422865
H	0.348884	0.825205	1.315207
H	2.696042	-1.830425	0.964699
H	2.279656	1.728895	-0.420615
H	2.262235	-0.358809	-1.695037
H	3.770358	-0.485697	-0.766236
N	1.876967	-2.834367	-0.630177
C	2.317626	-4.063578	-0.054936
O	2.862502	-4.058338	1.071802
N	2.123031	-5.210248	-0.758927
C	1.512933	-5.200873	-1.961759
N	1.348860	-6.374156	-2.591641
C	1.042779	-3.977778	-2.550497
C	1.238021	-2.831301	-1.844673
H	1.718015	-7.247692	-2.180017
H	0.927718	-6.394616	-3.511414
H	0.533873	-3.971515	-3.508042
H	0.894821	-1.866946	-2.200433
P	4.502542	0.960365	1.556682

O	5.130171	1.526372	0.295769
O	4.799674	1.666686	2.868111
O	4.931679	-0.615593	1.617625
C	4.798464	-1.410784	2.838972
C	6.074285	-2.211811	3.123852
O	6.173064	-3.420491	2.297234
C	7.137888	-3.227258	1.234535
C	7.391873	-1.452337	2.868187
C	7.729371	-1.811730	1.403138
H	8.166330	-1.827570	3.545713
H	3.954209	-2.098467	2.707768
H	4.614146	-0.745118	3.690752
H	5.992558	-2.562115	4.159646
H	7.895378	-4.010816	1.318073
H	7.276474	-0.372958	3.012133
H	7.240459	-1.112899	0.721206
H	8.803909	-1.817096	1.199598
N	6.485803	-3.452599	-0.061075
C	5.590645	-2.617500	-0.710440
N	4.997305	-3.176794	-1.749967
C	5.520195	-4.465639	-1.780374
C	5.250676	-5.578547	-2.606745
N	4.367415	-5.559086	-3.631294
N	5.937281	-6.724566	-2.352101
C	6.823892	-6.769687	-1.335867
N	7.142226	-5.785145	-0.481639
C	6.448284	-4.657169	-0.744074
H	5.421897	-1.616893	-0.345947
H	4.053651	-6.456288	-4.028058
H	3.739157	-4.765753	-3.690671
H	7.316143	-7.730280	-1.203349
H	6.287465	-14.692013	-5.409617
C	7.045331	-14.518218	-4.633217
O	7.263570	-13.078290	-4.463500
C	6.490654	-12.613535	-3.317432
C	6.527380	-15.008536	-3.283603
C	5.656381	-13.819808	-2.856290
O	7.694972	-15.215861	-2.421249
H	7.995808	-14.975928	-4.922062
H	7.178146	-12.283270	-2.537592
H	5.963520	-15.942723	-3.352500

H	4.708735	-13.879974	-3.401186
H	5.439047	-13.802850	-1.790028
N	5.703364	-11.430032	-3.694075
C	6.070198	-10.175562	-3.172871
O	7.049653	-10.000505	-2.440966
N	5.241692	-9.137211	-3.551151
C	4.176824	-9.186888	-4.442185
O	3.547128	-8.141988	-4.728633
C	3.891404	-10.499698	-5.005852
C	2.781034	-10.645578	-6.016358
C	4.667410	-11.543096	-4.606080
H	5.495464	-8.199593	-3.127422
H	2.970082	-10.010172	-6.892930
H	1.821319	-10.324493	-5.588745
H	2.693094	-11.686977	-6.346531
H	4.514090	-12.539632	-5.006368
P	7.556108	-15.387300	-0.791444
O	6.175107	-15.882461	-0.404988
O	8.748408	-16.220638	-0.353045
O	7.674803	-13.851901	-0.236020
C	8.963543	-13.160957	-0.299449
C	9.108207	-12.152144	0.840292
O	8.319481	-10.941722	0.597277
C	7.190915	-10.877369	1.501911
C	8.660037	-12.655991	2.228634
C	7.191661	-12.190371	2.312948
H	9.263942	-12.173037	3.004745
H	9.038565	-12.644268	-1.264624
H	9.773977	-13.895280	-0.211754
H	10.160764	-11.842639	0.838807
H	7.303541	-9.995680	2.139759
H	8.757046	-13.742600	2.321800
H	6.537053	-12.923084	1.835154
H	6.849449	-12.015595	3.337250
N	5.961006	-10.658580	0.740475
C	5.318610	-11.576023	-0.080589
N	4.266846	-11.078411	-0.698411
C	4.213212	-9.752433	-0.272735
C	3.301938	-8.706790	-0.597198
O	2.318233	-8.755157	-1.379995
N	3.606688	-7.511319	0.080028

C	4.646879	-7.344429	0.975507
N	4.730316	-6.141463	1.589585
N	5.521147	-8.313937	1.266288
C	5.260773	-9.472175	0.620029
H	5.700030	-12.581605	-0.168654
H	3.017810	-6.677914	-0.165823
H	4.096027	-5.371475	1.335061
H	5.603061	-5.897869	2.041238
Na	7.187039	-17.888227	0.678754
Na	6.116682	3.390167	1.594751

$Na_2d(CpG)_2$, Total Bonding Energy: -16625.24 kcal/mol

H	0.194616	0.725695	-0.559259
C	0.805547	0.459698	0.313836
O	0.804634	-0.999275	0.484700
C	2.100784	-1.520581	0.051105
C	2.264351	0.843466	0.068309
C	2.758757	-0.373978	-0.724244
O	2.905083	0.968929	1.383974
H	0.392479	0.917023	1.218037
H	2.693585	-1.781062	0.926616
H	2.377959	1.785145	-0.474802
H	2.368198	-0.301607	-1.744640
H	3.843445	-0.454514	-0.768691
N	1.904225	-2.767399	-0.692802
C	2.276446	-4.006462	-0.093028
O	2.750042	-4.014223	1.066052
N	2.095341	-5.148083	-0.807084
C	1.556284	-5.124316	-2.042357
N	1.406505	-6.292495	-2.685017
C	1.130554	-3.893220	-2.647750
C	1.317886	-2.751451	-1.934770
H	1.709588	-7.176128	-2.245106
H	1.002201	-6.303782	-3.612008
H	0.666677	-3.877266	-3.627656
H	1.011216	-1.780724	-2.305885
P	4.542352	0.996266	1.548123
O	5.200522	1.538799	0.293127
O	4.821277	1.716625	2.856204
O	4.949853	-0.584663	1.640160
C	4.741478	-1.354598	2.868084

C	5.953849	-2.235904	3.186880
O	5.984002	-3.454869	2.371844
C	7.004543	-3.355022	1.347331
C	7.325511	-1.570253	2.954168
C	7.668035	-1.970366	1.502206
H	8.057661	-1.989190	3.653017
H	3.854739	-1.985385	2.732739
H	4.588327	-0.666481	3.708015
H	5.826719	-2.567509	4.224373
H	7.713795	-4.174497	1.491197
H	7.281321	-0.483966	3.084497
H	7.224193	-1.260273	0.800857
H	8.743848	-2.034578	1.315683
N	6.402719	-3.583179	0.031621
C	5.571496	-2.720187	-0.674307
N	5.069438	-3.256257	-1.767792
C	5.580773	-4.552834	-1.783396
C	5.367471	-5.628602	-2.692612
O	4.655215	-5.636132	-3.729758
N	6.066543	-6.789564	-2.309900
C	6.902548	-6.891921	-1.213371
N	7.559334	-8.065529	-1.055774
N	7.101655	-5.889666	-0.351974
C	6.411613	-4.773801	-0.672888
H	5.383625	-1.725300	-0.302330
H	5.872104	-7.653748	-2.875622
H	7.359099	-8.868688	-1.667992
H	7.938813	-8.264791	-0.138441
H	5.891556	-15.078544	-5.412667
C	6.708674	-14.773953	-4.745150
O	6.843043	-13.311539	-4.772710
C	6.294718	-12.769112	-3.529845
C	6.361356	-15.127183	-3.299505
C	5.501569	-13.920208	-2.901044
O	7.626481	-15.200395	-2.556921
H	7.653083	-15.217209	-5.075349
H	7.113414	-12.467227	-2.878594
H	5.835416	-16.080681	-3.203003
H	4.516315	-14.032388	-3.365891
H	5.372214	-13.812017	-1.825585
N	5.534782	-11.550135	-3.818929

C	6.069139	-10.284359	-3.436373
O	7.186823	-10.229298	-2.874622
N	5.340283	-9.169528	-3.705838
C	4.151901	-9.244294	-4.337362
N	3.491718	-8.099650	-4.570673
C	3.615495	-10.503654	-4.772772
C	4.342910	-11.618468	-4.498084
H	3.881238	-7.196555	-4.257123
H	2.595627	-8.126566	-5.038874
H	2.674435	-10.561268	-5.308351
H	4.023528	-12.607683	-4.803968
P	7.666921	-15.175002	-0.911932
O	6.382412	-15.738987	-0.332424
O	8.971062	-15.844254	-0.514149
O	7.679869	-13.581060	-0.548123
C	8.898586	-12.779968	-0.679845
C	9.097851	-11.863741	0.532159
O	8.250876	-10.668842	0.467328
C	7.153094	-10.773412	1.408386
C	8.775370	-12.505721	1.897058
C	7.291556	-12.137464	2.116886
H	9.403993	-12.052558	2.671248
H	8.813266	-12.171844	-1.588284
H	9.766375	-13.446013	-0.753067
H	10.133239	-11.506060	0.478973
H	7.219566	-9.933630	2.105670
H	8.935991	-13.588852	1.888296
H	6.645047	-12.876472	1.638412
H	7.020863	-12.053605	3.173270
N	5.882195	-10.596607	0.701872
C	5.265946	-11.499441	-0.158502
N	4.201307	-11.007531	-0.757759
C	4.110894	-9.700578	-0.282105
C	3.193688	-8.656537	-0.595872
O	2.216906	-8.696383	-1.387736
N	3.489166	-7.468147	0.099103
C	4.510908	-7.314348	1.018339
N	4.578053	-6.121491	1.655425
N	5.381150	-8.286165	1.310787
C	5.146315	-9.427251	0.626410
H	5.677819	-12.487428	-0.290919

H	2.918809	-6.625675	-0.164428
H	3.964265	-5.342623	1.378545
H	5.449478	-5.882535	2.111990
Na	7.677095	-17.542012	0.802446
Na	6.162259	3.427594	1.606790

$Na_2d(GpA)_2$, Total Bonding Energy: -16713.40 kcal/mol

H	0.056448	0.589407	-0.223132
C	0.763722	0.388313	0.592801
O	0.872879	-1.060836	0.806508
C	2.105817	-1.532680	0.186093
C	2.166057	0.844980	0.192451
C	2.628263	-0.355524	-0.643826
O	2.927472	1.024520	1.435594
H	0.412769	0.846319	1.522103
H	2.816474	-1.810422	0.966553
H	2.171660	1.784811	-0.366177
H	2.120604	-0.316889	-1.614444
H	3.704473	-0.396069	-0.806848
N	1.857775	-2.747205	-0.571363
C	1.114763	-2.913963	-1.734206
N	1.111557	-4.160807	-2.169410
C	1.904155	-4.853198	-1.252566
C	2.286860	-6.224919	-1.187177
O	1.923534	-7.155776	-1.949530
N	3.177559	-6.476688	-0.123904
C	3.597996	-5.533333	0.796974
N	4.448023	-5.945427	1.774562
N	3.183406	-4.265966	0.782512
C	2.376064	-3.989327	-0.257002
H	0.601686	-2.081513	-2.196865
H	3.578121	-7.445659	-0.062838
H	4.947416	-6.838782	1.649506
H	4.972932	-5.184824	2.206552
P	4.570549	0.998256	1.489135
O	5.169509	1.474103	0.178024
O	4.960532	1.744507	2.753024
O	4.925856	-0.593702	1.603428
C	4.812097	-1.318259	2.868038
C	6.072018	-2.141802	3.145852
O	6.132577	-3.359059	2.329315

C	7.077185	-3.193640	1.242134
C	7.399756	-1.413189	2.857066
C	7.701095	-1.790482	1.388545
H	8.179691	-1.799445	3.522020
H	3.943304	-1.984180	2.804232
H	4.679452	-0.606299	3.691390
H	6.000905	-2.478805	4.186696
H	7.820255	-3.990593	1.325740
H	7.308955	-0.330661	2.995275
H	7.216101	-1.085491	0.710931
H	8.771327	-1.822489	1.166042
N	6.408896	-3.423637	-0.043887
C	5.600550	-2.563832	-0.772330
N	5.041453	-3.127628	-1.828799
C	5.491383	-4.443933	-1.788678
C	5.231190	-5.570457	-2.603224
N	4.408404	-5.556944	-3.678594
N	5.863396	-6.730316	-2.282577
C	6.672142	-6.785553	-1.204668
N	6.965875	-5.794910	-0.351200
C	6.344977	-4.645697	-0.691449
H	5.465368	-1.540358	-0.458645
H	4.117314	-6.459862	-4.073075
H	3.782294	-4.766634	-3.782229
H	7.124641	-7.757182	-1.021892
H	6.518233	-14.657384	-5.290266
C	7.274625	-14.527284	-4.503679
O	7.559551	-13.102157	-4.313380
C	6.750822	-12.597742	-3.216412
C	6.716589	-15.005368	-3.167399
C	5.891741	-13.785692	-2.737320
O	7.853907	-15.267147	-2.281904
H	8.206205	-15.024218	-4.787893
H	7.411721	-12.231128	-2.429809
H	6.112294	-15.912087	-3.253029
H	4.930580	-13.817760	-3.259958
H	5.699514	-13.764124	-1.667115
N	5.967532	-11.429797	-3.664868
C	6.281977	-10.155239	-3.155925
O	7.216550	-9.943796	-2.376211
N	5.444678	-9.146020	-3.589756

C	4.408941	-9.244479	-4.511592
O	3.751450	-8.226590	-4.825740
C	4.178225	-10.575950	-5.058897
C	3.099122	-10.773558	-6.093964
C	4.967703	-11.589217	-4.611389
H	5.610124	-8.199591	-3.135586
H	3.295132	-10.154344	-6.980279
H	2.121049	-10.466352	-5.698743
H	3.044974	-11.824320	-6.400132
H	4.852700	-12.598894	-4.991585
P	7.632032	-15.562407	-0.678140
O	6.221249	-16.044125	-0.400103
O	8.781316	-16.458022	-0.248325
O	7.763023	-14.077998	0.003517
C	9.067470	-13.413414	-0.015752
C	9.116175	-12.291733	1.015344
O	8.287474	-11.164036	0.581221
C	7.229141	-10.915715	1.537262
C	8.618091	-12.662806	2.432544
C	7.161223	-12.158793	2.444040
H	9.214901	-12.129958	3.180844
H	9.243262	-13.005346	-1.019314
H	9.850831	-14.143512	0.224160
H	10.156590	-11.942838	1.037174
H	7.449134	-10.010229	2.105323
H	8.688661	-13.738948	2.620418
H	6.498245	-12.910685	2.006623
H	6.798765	-11.897804	3.442997
N	5.985956	-10.638001	0.785547
C	5.372709	-9.354120	0.821629
O	5.820444	-8.473847	1.592812
N	4.303601	-9.128144	0.006817
C	3.821109	-10.098363	-0.796549
N	2.781743	-9.803258	-1.595115
C	4.396296	-11.411647	-0.811606
C	5.486713	-11.620389	-0.029371
H	2.460629	-8.824394	-1.703642
H	2.450199	-10.504903	-2.245116
H	4.004064	-12.188169	-1.457017
H	6.036890	-12.553373	-0.017249
Na	7.128559	-18.157123	0.583853

Na	6.348945	3.317045	1.378026

$Na_2d(GpC)_2$, Total Bonding Energy: -16625.64 kcal/mol

H	-0.050857	0.076987	0.020726
C	0.808848	0.162432	0.701450
O	1.318374	-1.163847	1.050254
C	2.285787	-1.562890	0.043957
C	1.972451	0.842389	-0.015090
C	2.518538	-0.326518	-0.851975
O	2.888308	1.330078	1.017741
H	0.512204	0.660931	1.627856
H	3.192659	-1.887151	0.560737
H	1.662205	1.686331	-0.637426
H	1.920674	-0.390412	-1.765816
H	3.562174	-0.204725	-1.137191
N	1.815583	-2.733042	-0.700132
C	0.879667	-2.800054	-1.729616
N	0.706913	-4.023603	-2.193208
C	1.571823	-4.811444	-1.433915
C	1.866150	-6.206572	-1.491624
O	1.352262	-7.067343	-2.250676
N	2.858152	-6.577834	-0.564240
C	3.467280	-5.715205	0.331594
N	4.374182	-6.245547	1.178034
N	3.182534	-4.412814	0.401874
C	2.261787	-4.027424	-0.499668
H	0.364637	-1.916797	-2.082426
H	3.150114	-7.585545	-0.556859
H	4.668317	-7.232758	1.113942
H	4.937581	-5.591673	1.708101
P	4.469987	1.656165	0.721250
O	4.701399	2.050120	-0.725072
O	4.901326	2.640190	1.795118
O	5.194536	0.200999	0.930763
C	5.190714	-0.386963	2.271928
C	6.287594	-1.437419	2.397403
O	5.939395	-2.630897	1.621928
C	6.925313	-2.859028	0.586093
C	7.683774	-0.997625	1.895675
C	7.739327	-1.558739	0.460918
H	8.458899	-1.457975	2.518150

H	4.209390	-0.842798	2.455570
H	5.372750	0.399548	3.015019
H	6.315619	-1.730056	3.454743
H	7.558948	-3.703122	0.862727
H	7.802703	0.090226	1.923603
H	7.252704	-0.869180	-0.234099
H	8.756655	-1.763154	0.112886
N	6.216649	-3.264319	-0.645551
C	6.328627	-4.589509	-1.145688
O	7.126963	-5.390238	-0.601081
N	5.562885	-4.946339	-2.213984
C	4.722992	-4.062928	-2.794067
N	3.991825	-4.479235	-3.840223
C	4.607327	-2.715768	-2.314600
C	5.352539	-2.374750	-1.231997
H	4.006872	-5.468485	-4.139191
H	3.297782	-3.858554	-4.237211
H	3.921414	-2.013320	-2.773201
H	5.293677	-1.402231	-0.758187
H	6.393886	-14.340308	-6.116721
C	7.018590	-14.412759	-5.214598
O	7.320674	-13.079264	-4.694283
C	6.265754	-12.693655	-3.773708
C	6.236735	-15.093700	-4.094659
C	5.356513	-13.930076	-3.610190
O	7.215742	-15.562276	-3.111764
H	7.966913	-14.903984	-5.447356
H	6.732117	-12.388327	-2.833619
H	5.644588	-15.946554	-4.437700
H	4.491446	-13.865848	-4.277323
H	4.994126	-14.053873	-2.590686
N	5.552969	-11.513298	-4.262539
C	4.600077	-11.423805	-5.274208
N	4.141174	-10.198136	-5.445085
C	4.823380	-9.433555	-4.498295
C	4.733177	-8.048348	-4.168439
O	4.013225	-7.176868	-4.719942
N	5.571076	-7.703418	-3.091432
C	6.416237	-8.582129	-2.433441
N	7.166004	-8.082021	-1.430789
N	6.524702	-9.872919	-2.756802

C	5.706392	-10.233037	-3.761162
H	4.298366	-12.293165	-5.842645
H	5.555568	-6.702463	-2.776325
H	7.082819	-7.101335	-1.119519
H	7.668853	-8.750703	-0.859648
P	6.820380	-15.886208	-1.551153
O	5.369500	-16.308344	-1.417171
O	7.881657	-16.846555	-1.042924
O	6.949500	-14.424328	-0.823181
C	8.274427	-13.812705	-0.702825
C	8.294302	-12.807849	0.443700
O	7.531670	-11.608549	0.087057
C	6.406302	-11.447152	0.983951
C	7.686438	-13.316073	1.772730
C	6.239999	-12.784224	1.727166
H	8.230417	-12.877650	2.616621
H	8.520061	-13.311769	-1.647826
H	9.019624	-14.591345	-0.497163
H	9.340760	-12.503107	0.570420
H	6.605077	-10.627692	1.676103
H	7.728186	-14.407352	1.848663
H	5.605243	-13.463982	1.152599
H	5.801049	-12.630544	2.717802
N	5.233454	-11.033706	0.184694
C	4.696814	-9.724363	0.308617
O	5.145075	-8.952760	1.191634
N	3.700441	-9.348452	-0.540204
C	3.218525	-10.201721	-1.468558
N	2.243974	-9.764893	-2.282467
C	3.727372	-11.537252	-1.591800
C	4.745010	-11.893690	-0.766048
H	1.935482	-8.778870	-2.259562
H	1.931884	-10.358644	-3.040491
H	3.344919	-12.216324	-2.344836
H	5.241231	-12.855082	-0.822643
Na	6.095878	-18.476583	-0.390021
Na	5.684182	4.225588	0.014082

$Na_2d(GpG)_2$, Total Bonding Energy: -16624.69 kcal/mol

H	0.053399	-0.235322	0.485141
C	0.928603	-0.053182	1.126465

O	1.582621	-1.314574	1.469579
C	2.514229	-1.647622	0.405654
C	1.986677	0.726785	0.349248
C	2.613342	-0.396703	-0.494302
O	2.883118	1.344144	1.329937
H	0.623214	0.429975	2.058185
H	3.464305	-1.913578	0.875041
H	1.563450	1.518062	-0.275582
H	2.004074	-0.506514	-1.394879
H	3.634168	-0.188087	-0.807005
N	2.080425	-2.844337	-0.321965
C	1.198662	-2.954514	-1.393899
N	1.051214	-4.194342	-1.820550
C	1.877338	-4.948860	-0.989501
C	2.161532	-6.344472	-0.974808
O	1.688913	-7.229411	-1.733865
N	3.085357	-6.685725	0.030932
C	3.623493	-5.796512	0.944720
N	4.425059	-6.314669	1.906528
N	3.357535	-4.489105	0.937908
C	2.513037	-4.129523	-0.047263
H	0.699698	-2.090617	-1.810108
H	3.388097	-7.689370	0.075684
H	4.763632	-7.286944	1.840159
H	5.005039	-5.646551	2.400908
P	4.458035	1.691033	1.026934
O	4.696019	2.043207	-0.430297
O	4.857621	2.726687	2.064663
O	5.208748	0.259426	1.292078
C	5.165450	-0.287472	2.650660
C	6.283960	-1.301527	2.861513
O	6.003025	-2.542107	2.134540
C	6.980588	-2.740241	1.084158
C	7.683354	-0.846076	2.389524
C	7.778587	-1.428461	0.966107
H	8.450315	-1.284377	3.037737
H	4.189208	-0.764015	2.806747
H	5.294660	0.526279	3.376078
H	6.278889	-1.546643	3.931082
H	7.620705	-3.588280	1.348178
H	7.784033	0.244107	2.402336

H	7.290950	-0.759397	0.252685
H	8.805118	-1.621501	0.640147
N	6.298220	-3.129088	-0.145940
C	5.525045	-2.320997	-0.971184
N	4.988840	-2.971963	-1.983705
C	5.421014	-4.287424	-1.818189
C	5.189914	-5.455131	-2.601857
O	4.508880	-5.558107	-3.655813
N	5.839460	-6.586107	-2.072066
C	6.631063	-6.590767	-0.937326
N	7.195099	-7.768043	-0.598551
N	6.850555	-5.500874	-0.194555
C	6.230644	-4.402835	-0.675618
H	5.409713	-1.269473	-0.751686
H	5.726375	-7.487334	-2.596989
H	7.145210	-8.597549	-1.207301
H	7.727987	-7.806699	0.259965
H	6.753346	-14.446834	-5.581373
C	7.447716	-14.395915	-4.730365
O	7.708414	-12.998695	-4.376761
C	6.797167	-12.599080	-3.316097
C	6.793149	-15.004919	-3.494686
C	5.924460	-13.835192	-3.012258
O	7.858162	-15.351915	-2.551196
H	8.400970	-14.862962	-4.992910
H	7.383435	-12.291738	-2.449881
H	6.205743	-15.899673	-3.716612
H	5.000234	-13.830684	-3.597253
H	5.659281	-13.914231	-1.960587
N	6.031746	-11.404459	-3.728989
C	6.313584	-10.134187	-3.138692
O	7.248208	-10.033514	-2.313320
N	5.541430	-9.072364	-3.495429
C	4.568927	-9.192854	-4.421886
N	3.834725	-8.109159	-4.710796
C	4.322554	-10.441762	-5.088788
C	5.081645	-11.505823	-4.716626
H	4.050226	-7.193404	-4.282728
H	3.117984	-8.166115	-5.423019
H	3.571689	-10.527681	-5.866167
H	4.973716	-12.478953	-5.181115

P	7.498982	-15.813296	-1.012295
O	6.084652	-16.353592	-0.923005
O	8.630222	-16.722679	-0.565546
O	7.515225	-14.400832	-0.179891
C	8.797305	-13.722514	0.023196
C	8.709037	-12.741894	1.187161
O	7.929449	-11.560586	0.803855
C	6.766768	-11.425618	1.654835
C	8.041927	-13.291501	2.471022
C	6.591538	-12.779982	2.366630
H	8.537043	-12.867867	3.351597
H	9.066557	-13.187008	-0.896375
H	9.569219	-14.467860	0.251346
H	9.735031	-12.405661	1.383351
H	6.917486	-10.610257	2.364569
H	8.095532	-14.383657	2.521508
H	5.996179	-13.457345	1.747652
H	6.102170	-12.655921	3.337624
N	5.622981	-11.028306	0.806458
C	4.990919	-9.764539	0.960873
O	5.338145	-9.008696	1.899582
N	4.011981	-9.421945	0.076857
C	3.630158	-10.267760	-0.903648
N	2.663011	-9.869124	-1.744737
C	4.231333	-11.561845	-1.051734
C	5.233323	-11.881747	-0.193716
H	2.305238	-8.898508	-1.719656
H	2.413868	-10.464724	-2.524331
H	3.920292	-12.238796	-1.838234
H	5.792708	-12.807130	-0.251724
Na	6.958235	-18.533752	-0.088938
Na	5.608563	4.258250	0.234140

$Na_2d(GpT)_2$, Total Bonding Energy: -16714.75 kcal/mol

H	0.055228	-0.828235	0.257281
C	0.851615	-0.523594	0.953349
O	1.692085	-1.665571	1.301638
C	2.647492	-1.851315	0.226605
C	1.805849	0.443836	0.256855
C	2.656952	-0.531146	-0.578482
O	2.529128	1.173535	1.302229

H	0.419516	-0.130324	1.876784
H	3.608559	-2.088952	0.687509
H	1.291350	1.175345	-0.372476
H	2.172734	-0.650871	-1.549872
H	3.666299	-0.171010	-0.756313
N	2.288698	-3.021128	-0.591149
C	1.353902	-3.128998	-1.618200
N	1.182825	-4.368474	-2.039358
C	2.043390	-5.128562	-1.248363
C	2.275774	-6.537562	-1.195719
O	1.718215	-7.436544	-1.873498
N	3.248247	-6.873723	-0.234951
C	3.859709	-5.979393	0.620738
N	4.717895	-6.492220	1.542041
N	3.614734	-4.669413	0.608054
C	2.727095	-4.309945	-0.338700
H	0.839162	-2.262033	-2.008675
H	3.523190	-7.884944	-0.160026
H	5.108004	-7.437329	1.390408
H	5.368956	-5.810377	1.918681
P	4.068414	1.719922	1.146952
O	4.379645	2.172499	-0.267704
O	4.250934	2.738057	2.261035
O	4.969831	0.379505	1.414047
C	4.819271	-0.283752	2.710823
C	5.906265	-1.333109	2.887145
O	5.686101	-2.425596	1.938878
C	6.845210	-2.579632	1.085712
C	7.355731	-0.843232	2.647397
C	7.648481	-1.273662	1.195773
H	8.033071	-1.353297	3.340875
H	3.829400	-0.755761	2.751440
H	4.905613	0.457707	3.515868
H	5.787697	-1.739229	3.900315
H	7.438592	-3.428893	1.430428
H	7.448217	0.238150	2.789122
H	7.276215	-0.518694	0.499710
H	8.710800	-1.450868	1.001808
N	6.377483	-2.937899	-0.267073
C	6.451353	-4.283638	-0.669699
O	7.020849	-5.163697	-0.012716

N	5.841738	-4.559805	-1.879502
C	5.201138	-3.654924	-2.718222
O	4.680796	-4.048048	-3.789217
C	5.185554	-2.273796	-2.266220
C	4.551706	-1.230389	-3.150582
C	5.740794	-1.995272	-1.056033
H	5.842039	-5.581115	-2.161994
H	5.022397	-1.239252	-4.142598
H	4.658666	-0.230837	-2.718203
H	3.484283	-1.440041	-3.300802
H	5.700513	-0.998277	-0.632691
H	6.299032	-13.513155	-5.522878
C	6.953443	-13.829641	-4.696379
O	7.324031	-12.684018	-3.870082
C	6.223469	-12.420983	-2.966604
C	6.183616	-14.741909	-3.744211
C	5.364191	-13.712051	-2.942805
O	7.172492	-15.496856	-2.969698
H	7.872360	-14.272857	-5.087406
H	6.657431	-12.162174	-1.998663
H	5.540040	-15.458275	-4.262611
H	4.409948	-13.568719	-3.454647
H	5.145245	-14.043038	-1.931687
N	5.462440	-11.237010	-3.401289
C	4.467247	-11.152212	-4.364813
N	4.047422	-9.917410	-4.586726
C	4.812921	-9.138793	-3.724806
C	4.874015	-7.739905	-3.509701
N	4.124945	-6.848581	-4.181462
N	5.764336	-7.290020	-2.581638
C	6.553668	-8.170793	-1.931809
N	6.606991	-9.501814	-2.083547
C	5.704731	-9.932305	-2.989311
H	4.092957	-12.031838	-4.869762
H	4.253637	-5.840889	-4.025205
H	3.435411	-7.176325	-4.844639
H	7.222349	-7.730025	-1.195793
P	6.946500	-16.009412	-1.428116
O	5.503578	-16.391902	-1.152971
O	8.006043	-17.072850	-1.193552
O	7.245591	-14.666085	-0.538786

C	8.570020	-14.054483	-0.669077
C	8.757566	-12.975933	0.387590
O	7.863497	-11.850694	0.105974
C	6.967348	-11.630319	1.225977
C	8.462297	-13.401053	1.846422
C	7.022662	-12.903735	2.087630
H	9.160109	-12.894395	2.521944
H	8.657581	-13.617311	-1.671701
H	9.342996	-14.822163	-0.534282
H	9.789349	-12.616140	0.281138
H	7.300484	-10.757521	1.789332
H	8.558156	-14.483026	1.981917
H	6.304480	-13.648666	1.736909
H	6.814607	-12.673088	3.136829
N	5.637081	-11.284058	0.686924
C	5.195157	-9.929245	0.687918
O	5.856276	-9.065325	1.308420
N	4.051668	-9.619628	0.016060
C	3.356085	-10.563025	-0.649388
N	2.270696	-10.180179	-1.343083
C	3.758363	-11.939367	-0.628331
C	4.909749	-12.241130	0.026069
H	2.069439	-9.178319	-1.500368
H	1.776629	-10.866071	-1.899791
H	3.197164	-12.697907	-1.161156
H	5.333679	-13.237386	0.026415
Na	6.228430	-18.690485	-0.495506
Na	4.995257	4.441021	0.594206

Na_2d$(TpA)_2$, Total Bonding Energy: -16799.85 kcal/mol

H	-0.053275	0.104351	0.380155
C	0.781585	0.058182	1.093775
O	1.130272	-1.338034	1.368643
C	2.224653	-1.733723	0.495073
C	2.032761	0.666270	0.466420
C	2.533151	-0.508016	-0.385603
O	2.939002	1.028161	1.559423
H	0.497958	0.528927	2.039088
H	3.079694	-2.016659	1.110522
H	1.823302	1.557843	-0.130630
H	1.951383	-0.525480	-1.312606

H	3.586050	-0.431274	-0.648258
N	1.853426	-2.941155	-0.265342
C	2.479497	-4.163235	0.042661
O	3.277275	-4.306016	0.973993
N	2.129899	-5.208184	-0.793100
C	1.176966	-5.195184	-1.803999
O	0.949422	-6.234995	-2.467982
C	0.490233	-3.927948	-2.007415
C	-0.600368	-3.831787	-3.045405
C	0.864149	-2.875531	-1.230376
H	2.627292	-6.123804	-0.589369
H	-1.410001	-4.540800	-2.823351
H	-0.214124	-4.087371	-4.041605
H	-1.015807	-2.818155	-3.075034
H	0.381157	-1.909498	-1.330191
P	4.529691	1.352259	1.295048
O	4.773175	1.798202	-0.134201
O	4.957662	2.295172	2.406683
O	5.243300	-0.112950	1.456868
C	5.307255	-0.724736	2.785304
C	6.506073	-1.663463	2.900948
O	6.276697	-2.915494	2.173704
C	7.141925	-2.995947	1.017416
C	7.836237	-1.107543	2.348444
C	7.863362	-1.638577	0.900784
H	8.672115	-1.517904	2.925505
H	4.378557	-1.282094	2.962779
H	5.412711	0.061671	3.543251
H	6.585389	-1.928085	3.962787
H	7.841501	-3.826049	1.156365
H	7.872916	-0.014190	2.390548
H	7.298016	-0.971920	0.245190
H	8.874245	-1.765692	0.502261
N	6.347197	-3.344220	-0.161300
C	5.441383	-2.533930	-0.821305
N	4.802930	-3.142514	-1.805811
C	5.313183	-4.437128	-1.787354
C	5.047285	-5.576582	-2.581590
N	4.143631	-5.604610	-3.580532
N	5.764537	-6.702641	-2.316385
C	6.658659	-6.710684	-1.308961

N	6.973679	-5.701259	-0.481158
C	6.270602	-4.584614	-0.770606
H	5.308172	-1.508306	-0.511179
H	3.942399	-6.487524	-4.067218
H	3.545994	-4.801803	-3.726638
H	7.169212	-7.659356	-1.160144
H	6.780380	-14.427082	-5.676546
C	7.424782	-14.344488	-4.789788
O	7.634492	-12.934487	-4.451353
C	6.675260	-12.544593	-3.428932
C	6.717760	-14.945997	-3.578457
C	5.801350	-13.785618	-3.168203
O	7.745655	-15.260024	-2.582553
H	8.401448	-14.794467	-4.988622
H	7.220275	-12.237844	-2.535267
H	6.161374	-15.857093	-3.814687
H	4.923021	-13.798987	-3.820690
H	5.459304	-13.852021	-2.137703
N	5.918950	-11.359223	-3.872452
C	6.151408	-10.123336	-3.240957
O	7.014499	-9.951505	-2.374793
N	5.325435	-9.100542	-3.669298
C	4.389589	-9.147711	-4.694970
O	3.725819	-8.125012	-4.989450
C	4.261031	-10.429294	-5.373382
C	3.305853	-10.562796	-6.533402
C	5.030473	-11.459314	-4.928557
H	5.473045	-8.174770	-3.171166
H	3.570049	-9.860045	-7.335759
H	2.279693	-10.323643	-6.222215
H	3.326797	-11.582435	-6.934429
H	4.986988	-12.433796	-5.403190
P	7.360150	-15.578457	-1.015246
O	5.930310	-16.069215	-0.888385
O	8.461615	-16.480968	-0.485713
O	7.413824	-14.103293	-0.306289
C	8.713123	-13.453030	-0.123119
C	8.689020	-12.504064	1.072962
O	7.956834	-11.272419	0.763549
C	6.722376	-11.214320	1.515794
C	8.024726	-13.065400	2.348726

C	6.568787	-12.570841	2.231567
H	8.508308	-12.635126	3.232655
H	8.960915	-12.896781	-1.035990
H	9.479071	-14.217048	0.060434
H	9.731033	-12.212383	1.251954
H	6.775975	-10.378294	2.220127
H	8.089985	-14.157099	2.398877
H	5.988535	-13.256744	1.609781
H	6.070241	-12.449374	3.197675
N	5.616736	-10.895793	0.611736
C	5.054557	-11.726467	-0.340625
N	4.122543	-11.141596	-1.073087
C	4.074587	-9.841579	-0.578896
C	3.288724	-8.719108	-0.928435
N	2.373013	-8.719617	-1.916822
N	3.471163	-7.580442	-0.205354
C	4.394747	-7.545217	0.774768
N	5.206038	-8.537224	1.176109
C	4.998093	-9.666421	0.464428
H	5.392886	-12.747587	-0.433904
H	1.888888	-7.848717	-2.170433
H	2.294284	-9.531994	-2.514190
H	4.483039	-6.587885	1.283324
Na	6.744442	-18.192788	0.140034
Na	5.720889	3.952890	0.694001

Nad(ApA), Total Bonding Energy: -8531.40 kcal/mol

H	0.610455	-0.065680	-1.156789
C	1.053820	0.026300	-0.154833
O	1.183990	-1.296702	0.461924
C	2.523087	-1.806964	0.200309
C	2.479427	0.555412	-0.273794
C	3.248252	-0.724965	-0.619731
O	2.868793	1.097935	1.032175
H	0.414236	0.637942	0.487731
H	3.016227	-1.998113	1.154384
H	2.584827	1.335198	-1.032254
H	3.150734	-0.915371	-1.692697
H	4.302408	-0.682583	-0.355337
N	2.460774	-3.103433	-0.470706
C	2.135428	-3.368166	-1.793789

N	2.121780	-4.655286	-2.089168
C	2.459683	-5.281387	-0.892439
C	2.650059	-6.637875	-0.546243
N	2.524262	-7.644100	-1.458116
N	2.943651	-6.941631	0.743654
C	3.055656	-5.929390	1.634113
N	2.936908	-4.608483	1.418527
C	2.651013	-4.338608	0.130306
H	1.916912	-2.566610	-2.486457
H	2.870801	-8.558646	-1.185946
H	2.655949	-7.393782	-2.433390
H	3.274795	-6.229498	2.656980
P	4.336457	1.814991	1.221846
O	4.738643	2.587848	-0.021137
O	4.251701	2.579347	2.532044
O	5.382217	0.560787	1.336516
C	5.214037	-0.384261	2.438430
C	6.095569	-1.602686	2.209135
O	5.569061	-2.368265	1.077673
C	6.674169	-2.732889	0.220122
C	7.583502	-1.292032	1.873365
C	7.682415	-1.587342	0.363227
H	8.237604	-1.966069	2.436494
H	4.163491	-0.693475	2.501169
H	5.502328	0.100451	3.379709
H	6.019284	-2.222860	3.112647
H	7.102689	-3.687783	0.548324
H	7.846248	-0.257810	2.115216
H	7.342455	-0.723042	-0.216044
H	8.685130	-1.878932	0.037097
N	6.167806	-2.960502	-1.125531
C	5.837589	-2.017013	-2.084327
N	5.319916	-2.536924	-3.183930
C	5.308613	-3.907565	-2.938509
C	4.876549	-5.010029	-3.712049
N	4.370168	-4.865601	-4.960720
N	5.005026	-6.255748	-3.187196
C	5.542548	-6.381847	-1.953893
N	5.977783	-5.417405	-1.124529
C	5.827864	-4.192702	-1.668217
H	6.004449	-0.963901	-1.911590

H	3.932938	-5.661417	-5.410578
H	4.130562	-3.939818	-5.292942
H	5.627355	-7.401769	-1.585046
Na	4.926770	4.680492	1.349625

Nad(ApC), Total Bonding Energy: -8134.89 kcal/mol

H	6.292536	-13.836244	-5.675955
C	6.995066	-14.047600	-4.856415
O	7.367796	-12.811287	-4.170269
C	6.353550	-12.521127	-3.178549
C	6.302548	-14.883972	-3.784071
C	5.494411	-13.803432	-3.043335
O	7.348351	-15.521937	-2.982808
H	7.908979	-14.502335	-5.246774
H	6.866177	-12.248359	-2.252001
H	5.657381	-15.663367	-4.199093
H	4.534838	-13.699767	-3.554130
H	5.290299	-14.060946	-2.006704
N	5.560565	-11.339457	-3.555148
C	4.421423	-11.268144	-4.345009
N	3.894195	-10.057074	-4.409198
C	4.735017	-9.278529	-3.618828
C	4.699908	-7.914127	-3.247035
N	3.699571	-7.083770	-3.631275
N	5.689648	-7.438080	-2.447625
C	6.649846	-8.294718	-2.033322
N	6.776832	-9.605724	-2.299966
C	5.781300	-10.048694	-3.091650
H	4.027163	-12.138104	-4.851915
H	3.786358	-6.089395	-3.456394
H	3.042924	-7.391059	-4.337774
H	7.421175	-7.859382	-1.401220
P	7.081653	-16.062022	-1.454512
O	5.644504	-16.507052	-1.255001
O	8.175434	-17.078493	-1.176651
O	7.282259	-14.711915	-0.546593
C	8.607521	-14.088903	-0.565621
C	8.694439	-13.007523	0.499447
O	7.843499	-11.877300	0.115572
C	6.929761	-11.566836	1.199017
C	8.238327	-13.433622	1.919494

C	6.814460	-12.855663	2.029361
H	8.895072	-12.978903	2.668898
H	8.782093	-13.655441	-1.558655
H	9.371364	-14.850222	-0.361668
H	9.735700	-12.659448	0.507266
H	7.323519	-10.741833	1.796390
H	8.258709	-14.521132	2.041402
H	6.092619	-13.538691	1.571768
H	6.507978	-12.641483	3.058041
N	5.668128	-11.087696	0.610063
C	5.247417	-9.729214	0.775334
O	5.910523	-8.958661	1.502780
N	4.114085	-9.325388	0.128715
C	3.434452	-10.174356	-0.655920
N	2.326196	-9.710482	-1.282339
C	3.828076	-11.543260	-0.832749
C	4.955654	-11.943182	-0.187629
H	2.144535	-8.712943	-1.279090
H	1.893110	-10.264912	-2.010164
H	3.271394	-12.219221	-1.471842
H	5.369003	-12.939753	-0.283333
Na	6.442307	-18.780307	-0.576125

Nad(ApG), Total Bonding Energy: -8695.83 kcal/mol

H	1.075345	-0.851244	-1.199730
C	1.290340	-0.487332	-0.184572
O	0.979959	-1.532839	0.796730
C	2.137164	-2.364327	0.958049
C	2.787291	-0.228016	-0.039879
C	3.323265	-1.628010	0.288557
O	3.005000	0.717118	1.058559
H	0.667918	0.380698	0.045745
H	2.287885	-2.564497	2.020898
H	3.224271	0.177159	-0.956838
H	3.572034	-2.132395	-0.647996
H	4.213194	-1.615840	0.915753
N	1.917840	-3.686177	0.327091
C	1.416534	-3.922589	-0.941580
N	1.535290	-5.182227	-1.333318
C	2.174667	-5.806156	-0.267029
C	2.628846	-7.128054	-0.068954

N	2.540397	-8.079123	-1.042956
N	3.243526	-7.435033	1.100597
C	3.401218	-6.457859	2.022761
N	3.033324	-5.166899	1.944795
C	2.423029	-4.893490	0.774338
H	0.960982	-3.126454	-1.515750
H	2.693293	-9.046719	-0.777567
H	1.899953	-7.914012	-1.811664
H	3.892582	-6.760662	2.944949
P	4.409035	1.573573	1.101250
O	4.781262	2.097385	-0.274033
O	4.235089	2.586626	2.219332
O	5.548497	0.461794	1.470400
C	5.501878	-0.199343	2.769748
C	6.578320	-1.278172	2.829191
O	6.301941	-2.334053	1.851390
C	7.228157	-2.246016	0.740745
C	8.015747	-0.813271	2.518372
C	8.149122	-1.037681	0.996131
H	8.723677	-1.447358	3.063036
H	4.514434	-0.658044	2.911015
H	5.673368	0.537766	3.564722
H	6.507285	-1.737426	3.822630
H	7.782825	-3.187374	0.703993
H	8.182154	0.231476	2.801044
H	7.794193	-0.157928	0.457970
H	9.173084	-1.260773	0.683323
N	6.486149	-2.185775	-0.529725
C	6.070851	-1.084978	-1.274610
N	5.369401	-1.417023	-2.341773
C	5.296256	-2.806701	-2.296293
C	4.593933	-3.727557	-3.134590
O	3.906454	-3.500529	-4.149759
N	4.742874	-5.057729	-2.657717
C	5.467365	-5.444779	-1.546948
N	5.538794	-6.784380	-1.292172
N	6.108894	-4.582414	-0.767194
C	5.980117	-3.298013	-1.174888
H	6.280061	-0.073015	-0.960759
H	4.259250	-5.770913	-3.198869
H	4.796249	-7.379347	-1.652898

H	5.852725	-7.026391	-0.357576
Na	4.782271	4.434614	0.610858

Nad(ApT), Total Bonding Energy: -8393.14 kcal/mol

H	5.548618	-0.641802	1.632273
C	6.390106	-1.069940	2.197194
O	6.571275	-2.480143	1.856003
C	7.373765	-2.552708	0.652234
C	7.692940	-0.403777	1.765223
C	7.981205	-1.142875	0.446730
O	8.679981	-0.641458	2.819982
H	6.197845	-1.007426	3.271175
H	8.129973	-3.326843	0.802127
H	7.594966	0.674929	1.616126
H	7.481498	-0.603133	-0.360043
H	9.041436	-1.185797	0.211642
N	6.567821	-2.999701	-0.495475
C	5.749488	-2.249732	-1.328255
N	5.173398	-2.957116	-2.285160
C	5.632402	-4.253728	-2.071486
C	5.409098	-5.470678	-2.759006
N	4.645979	-5.544569	-3.874599
N	6.005334	-6.597611	-2.285953
C	6.777263	-6.502302	-1.180378
N	7.071970	-5.409705	-0.455413
C	6.481490	-4.306898	-0.956544
H	5.615992	-1.187174	-1.180158
H	4.414704	-6.450417	-4.265045
H	4.110759	-4.738304	-4.170705
H	7.219414	-7.435350	-0.838375
P	10.294689	-0.504195	2.547736
O	10.597398	0.511220	1.461603
O	10.929679	-0.286453	3.910757
O	10.693926	-1.971340	1.936989
C	10.470780	-3.133284	2.797073
C	10.978143	-4.391927	2.114122
O	10.120898	-4.690504	0.961447
C	10.950557	-4.898363	-0.205022
C	12.430680	-4.321582	1.570467
C	12.243685	-4.111189	0.054875
H	12.942120	-5.270441	1.763750

H	9.396744	-3.226255	3.003271
H	11.011012	-2.997417	3.742788
H	10.869342	-5.208988	2.839367
H	11.163138	-5.963028	-0.326776
H	12.997668	-3.510141	2.036765
H	12.103713	-3.048858	-0.162933
H	13.073149	-4.498338	-0.544670
N	10.182834	-4.494839	-1.396343
C	9.692659	-5.483717	-2.258258
O	9.900765	-6.693580	-2.119452
N	8.945149	-4.986377	-3.315695
C	8.642209	-3.647066	-3.612426
O	7.996204	-3.369048	-4.636494
C	9.149364	-2.686358	-2.641800
C	8.854167	-1.223005	-2.847299
C	9.880888	-3.156971	-1.597637
H	8.590016	-5.688273	-3.962017
H	9.208444	-0.895818	-3.833588
H	9.338001	-0.615502	-2.076564
H	7.771539	-1.039777	-2.818027
H	10.276245	-2.491552	-0.840310
Na	12.035874	1.793831	3.048381

Nad(CpA), Total Bonding Energy: -8135.22 kcal/mol

H	1.411979	-0.513276	-1.972151
C	1.490665	-0.299319	-0.897574
O	0.884199	-1.403549	-0.139104
C	1.946336	-2.192221	0.468180
C	2.962398	-0.257703	-0.482016
C	3.240315	-1.736499	-0.212626
O	3.069513	0.529005	0.752968
H	0.954557	0.623583	-0.656474
H	1.967202	-2.008140	1.543047
H	3.609336	0.173866	-1.250275
H	3.368181	-2.252058	-1.171792
H	4.114465	-1.924620	0.405266
N	1.670030	-3.629579	0.300872
C	1.611363	-4.496090	1.441793
O	1.733928	-4.017997	2.589055
N	1.430138	-5.832896	1.221081
C	1.296349	-6.309295	-0.028147

N	1.129542	-7.640169	-0.183705
C	1.329417	-5.459965	-1.186381
C	1.521163	-4.132098	-0.967097
H	1.088542	-8.245722	0.627612
H	0.991806	-8.041900	-1.101776
H	1.208769	-5.858729	-2.187150
H	1.554810	-3.403824	-1.770211
P	4.533313	1.094673	1.239936
O	5.307116	1.695705	0.080449
O	4.245759	1.986364	2.435387
O	5.368078	-0.240984	1.674125
C	4.902327	-1.074831	2.775473
C	5.795274	-2.308416	2.882357
O	5.659170	-3.154757	1.692866
C	6.832306	-3.033956	0.849111
C	7.308042	-2.031978	2.998434
C	7.795914	-2.044312	1.532148
H	7.778119	-2.842719	3.565349
H	3.866506	-1.385851	2.588818
H	4.945413	-0.506450	3.713479
H	5.422892	-2.894805	3.730723
H	7.264503	-4.033073	0.749165
H	7.515992	-1.079845	3.497677
H	7.700768	-1.048178	1.098280
H	8.830836	-2.382240	1.429117
N	6.430506	-2.650101	-0.514240
C	6.296716	-1.390480	-1.083040
N	5.808102	-1.415732	-2.311760
C	5.590636	-2.765964	-2.567498
C	5.060273	-3.452563	-3.685339
N	4.633136	-2.807483	-4.798210
N	4.948333	-4.805775	-3.616949
C	5.353217	-5.429833	-2.488974
N	5.870290	-4.889354	-1.370511
C	5.964080	-3.546834	-1.464190
H	6.532926	-0.489312	-0.537007
H	4.390977	-3.347253	-5.621160
H	4.848389	-1.825172	-4.916007
H	5.246701	-6.512757	-2.490784
Na	5.405959	3.889717	1.284210

Nad(CpC), Total Bonding Energy: -7738.30 kcal/mol

H	6.560890	-15.098784	-5.657082
C	7.276858	-14.859452	-4.860407
O	7.458885	-13.400990	-4.801234
C	6.847962	-12.911239	-3.567087
C	6.707380	-15.266358	-3.499649
C	5.883579	-14.023349	-3.136373
O	7.845476	-15.468071	-2.593786
H	8.243757	-15.329380	-5.066093
H	7.624191	-12.772309	-2.815164
H	6.113065	-16.182451	-3.536235
H	4.971396	-14.027899	-3.741940
H	5.611714	-13.975210	-2.083121
N	6.257002	-11.591845	-3.786890
C	6.929621	-10.424418	-3.276757
O	8.059027	-10.545661	-2.764346
N	6.287688	-9.223685	-3.373913
C	5.108837	-9.128599	-4.008550
N	4.510080	-7.920804	-4.056926
C	4.479180	-10.256322	-4.640414
C	5.091905	-11.461126	-4.500940
H	4.939443	-7.121456	-3.606191
H	3.621217	-7.799194	-4.523578
H	3.556312	-10.153160	-5.200170
H	4.690129	-12.368123	-4.936573
P	7.613053	-15.627323	-0.971827
O	6.258854	-16.248681	-0.686356
O	8.844510	-16.336229	-0.437770
O	7.537016	-14.082611	-0.434781
C	8.748796	-13.287891	-0.226026
C	8.605938	-12.418442	1.021768
O	7.716299	-11.279960	0.762301
C	6.533198	-11.359732	1.594265
C	8.012062	-13.135965	2.255713
C	6.512317	-12.783388	2.180206
H	8.458528	-12.727281	3.168704
H	8.903947	-12.649586	-1.104453
H	9.607463	-13.956027	-0.091507
H	9.600634	-12.006118	1.231274
H	6.576392	-10.597385	2.373706
H	8.188635	-14.215842	2.221213

H	6.000044	-13.462124	1.491950
H	6.010080	-12.803495	3.152392
N	5.358102	-11.025741	0.757189
C	4.526892	-9.903337	1.060291
O	4.739903	-9.232778	2.095506
N	3.512570	-9.601010	0.193676
C	3.295695	-10.357490	-0.895461
N	2.281690	-10.010737	-1.718142
C	4.095605	-11.508008	-1.204255
C	5.121125	-11.788332	-0.357262
H	1.737056	-9.177195	-1.531633
H	2.120591	-10.516687	-2.578773
H	3.903164	-12.111897	-2.082526
H	5.812664	-12.608214	-0.511407
Na	7.376608	-18.157665	0.472194

Nad(CpG), Total Bonding Energy: -8300.86 kcal/mol

H	1.464921	-0.634357	-1.774651
C	1.533547	-0.400993	-0.702189
O	1.011579	-1.521793	0.086660
C	2.103906	-2.400359	0.440297
C	3.000389	-0.262700	-0.302304
C	3.394441	-1.723236	-0.064094
O	3.086245	0.538919	0.921149
H	0.932196	0.482265	-0.471108
H	2.091858	-2.565015	1.516966
H	3.600331	0.210917	-1.084816
H	3.684303	-2.157188	-1.025280
H	4.220762	-1.848722	0.632644
N	1.920158	-3.740636	-0.183584
C	2.063707	-4.933412	0.590485
O	2.213391	-4.857522	1.827521
N	2.037093	-6.132603	-0.073041
C	1.861776	-6.173049	-1.399748
N	1.934242	-7.385610	-2.015609
C	1.658151	-4.995824	-2.191385
C	1.709612	-3.804298	-1.535608
H	1.901083	-8.220996	-1.440559
H	1.608599	-7.472674	-2.971103
H	1.491542	-5.048675	-3.261298
H	1.585290	-2.853223	-2.040976

P	4.534127	1.198686	1.340224
O	5.252090	1.775961	0.134481
O	4.233128	2.135028	2.498234
O	5.434672	-0.077499	1.822289
C	5.001948	-0.851285	2.981289
C	5.934608	-2.041687	3.176224
O	5.812571	-2.978388	2.055634
C	6.988125	-2.909128	1.211984
C	7.439156	-1.712261	3.257376
C	7.914459	-1.824620	1.791655
H	7.938027	-2.462224	3.880605
H	3.978407	-1.212264	2.816421
H	5.021858	-0.216321	3.876324
H	5.586045	-2.570028	4.071812
H	7.455274	-3.897778	1.220192
H	7.622367	-0.718328	3.678766
H	7.766219	-0.872191	1.281651
H	8.962744	-2.122702	1.701572
N	6.579660	-2.683414	-0.184738
C	6.490594	-1.503423	-0.918245
N	6.000575	-1.682994	-2.130173
C	5.728410	-3.046574	-2.200931
C	5.105596	-3.818135	-3.230942
O	4.695599	-3.454536	-4.350411
N	4.956595	-5.172376	-2.825647
C	5.344911	-5.704507	-1.610978
N	5.144367	-7.043797	-1.433495
N	5.919186	-4.980272	-0.657989
C	6.075553	-3.679028	-0.998921
H	6.766890	-0.548421	-0.496511
H	4.523336	-5.786490	-3.511578
H	4.385624	-7.476306	-1.955438
H	5.229194	-7.364382	-0.473858
Na	5.301192	4.020864	1.233509

Nad(CpT), Total Bonding Energy: -7995.24 kcal/mol

H	6.299586	-15.059290	-5.162138
C	7.195235	-14.813639	-4.578312
O	7.428880	-13.364375	-4.668051
C	7.268051	-12.793739	-3.333714
C	6.962352	-15.127862	-3.095557

C	6.399418	-13.802681	-2.575210
O	8.263013	-15.420446	-2.478662
H	8.065057	-15.343831	-4.980169
H	8.245503	-12.695867	-2.862374
H	6.295812	-15.980767	-2.944008
H	5.346272	-13.717856	-2.863916
H	6.502513	-13.684755	-1.499483
N	6.725297	-11.439183	-3.423499
C	7.548541	-10.313364	-3.073911
O	8.764202	-10.480613	-2.854966
N	6.949976	-9.087832	-2.994757
C	5.666550	-8.930382	-3.354646
N	5.115061	-7.704670	-3.223351
C	4.868202	-10.012486	-3.858941
C	5.436180	-11.247156	-3.852520
H	5.672860	-6.929350	-2.885693
H	4.169610	-7.525290	-3.533800
H	3.845473	-9.862512	-4.185927
H	4.906805	-12.132508	-4.186148
P	8.330575	-15.940783	-0.917848
O	7.198252	-16.904684	-0.616177
O	9.753624	-16.432168	-0.716101
O	8.037061	-14.601323	-0.022981
C	9.002464	-13.506006	-0.063947
C	8.442235	-12.293826	0.661806
O	7.338794	-11.730617	-0.115416
C	6.375120	-11.220491	0.829792
C	7.880044	-12.565627	2.094592
C	6.373366	-12.241424	1.975939
H	8.363477	-11.897148	2.814088
H	9.223438	-13.243479	-1.105024
H	9.929450	-13.821482	0.430077
H	9.250174	-11.549539	0.702527
H	6.676137	-10.232708	1.190573
H	8.051750	-13.601232	2.401251
H	5.808416	-13.132543	1.683506
H	5.944229	-11.826273	2.893212
N	5.101238	-11.036339	0.117789
C	4.601512	-9.742082	-0.079755
O	5.110824	-8.720171	0.391593
N	3.453173	-9.698379	-0.857327

C	2.787099	-10.764140	-1.483200
O	1.789371	-10.541801	-2.191058
C	3.367127	-12.073245	-1.225007
C	2.740979	-13.291259	-1.853989
C	4.474502	-12.138566	-0.440921
H	3.070858	-8.767212	-1.009369
H	1.693535	-13.398596	-1.542793
H	3.288139	-14.197222	-1.571623
H	2.740730	-13.201498	-2.949454
H	4.948912	-13.085269	-0.218522
Na	8.875388	-18.650035	0.042751

Nad(GpA), Total Bonding Energy: -8696.62 kcal/mol

H	0.089939	0.651787	-0.357245
C	0.764054	0.400228	0.471973
O	0.794898	-1.060262	0.649114
C	2.066083	-1.559740	0.140904
C	2.197745	0.803130	0.123827
C	2.649046	-0.415392	-0.691556
O	2.922060	0.951730	1.394226
H	0.409250	0.854480	1.401893
H	2.723085	-1.803593	0.977900
H	2.258819	1.742818	-0.430935
H	2.168332	-0.376362	-1.676063
H	3.727019	-0.490464	-0.821590
N	1.865525	-2.805674	-0.579797
C	1.175457	-3.030286	-1.763919
N	1.231009	-4.288799	-2.162271
C	2.007813	-4.928263	-1.197003
C	2.438204	-6.287325	-1.088153
O	2.173940	-7.260305	-1.820928
N	3.295865	-6.455357	0.037138
C	3.661744	-5.470438	0.935603
N	4.504578	-5.811839	1.957270
N	3.205886	-4.227387	0.858800
C	2.413866	-4.018210	-0.212257
H	0.650656	-2.230233	-2.268646
H	3.682758	-7.389432	0.150180
H	5.059087	-6.652189	1.825878
H	5.045892	-5.012951	2.305289
P	4.562839	0.896910	1.495822

O	5.206034	1.371871	0.205173
O	4.929417	1.624683	2.777063
O	4.890912	-0.700059	1.603126
C	4.788769	-1.436935	2.859697
C	6.078777	-2.208015	3.148081
O	6.197723	-3.418720	2.326642
C	7.103399	-3.198082	1.215940
C	7.381586	-1.432707	2.872160
C	7.716503	-1.792184	1.404226
H	8.167432	-1.790700	3.545487
H	3.952656	-2.140165	2.770343
H	4.611237	-0.739824	3.687112
H	6.012765	-2.556887	4.184997
H	7.854420	-3.990592	1.250292
H	7.249525	-0.354446	3.010240
H	7.253864	-1.073252	0.726772
H	8.792169	-1.818904	1.210502
N	6.402254	-3.386527	-0.062913
C	5.659970	-2.473635	-0.798148
N	5.068417	-2.996151	-1.857699
C	5.430044	-4.338914	-1.819182
C	5.104604	-5.436411	-2.647583
N	4.289900	-5.312936	-3.732632
N	5.644111	-6.648465	-2.356675
C	6.441263	-6.754514	-1.271447
N	6.791176	-5.797792	-0.391131
C	6.259134	-4.601705	-0.717169
H	5.596050	-1.441044	-0.493353
H	3.897616	-6.169153	-4.111593
H	3.684589	-4.498770	-3.764106
H	6.846989	-7.746654	-1.083722
Na	6.387729	3.178419	1.460628

Nad(GpC), Total Bonding Energy: -8299.29 kcal/mol

H	-0.149340	0.905156	-0.375985
C	0.598454	0.695513	0.399786
O	0.615048	-0.749004	0.682769
C	1.801517	-1.326111	0.080651
C	1.997660	1.031300	-0.115941
C	2.344110	-0.255863	-0.875335
O	2.853444	1.246617	1.056582

H	0.347387	1.224682	1.323690
H	2.531415	-1.567004	0.857996
H	2.021431	1.923426	-0.746485
H	1.783011	-0.270819	-1.817029
H	3.406992	-0.374369	-1.086888
N	1.477352	-2.589600	-0.568866
C	0.612863	-2.814163	-1.634641
N	0.655026	-4.057558	-2.077474
C	1.606736	-4.684061	-1.274262
C	2.143212	-6.008791	-1.319922
O	1.832213	-6.959545	-2.064322
N	3.184628	-6.164803	-0.363761
C	3.637352	-5.194614	0.512377
N	4.673806	-5.511678	1.338850
N	3.094495	-3.985222	0.584056
C	2.124020	-3.786423	-0.331111
H	-0.020787	-2.025565	-2.017664
H	3.648070	-7.071004	-0.370583
H	5.220490	-6.336766	1.117754
H	5.209979	-4.700316	1.663337
P	4.492300	1.297125	0.911732
O	4.893668	1.791453	-0.465031
O	5.000718	2.067857	2.116645
O	4.930026	-0.278818	0.986290
C	5.019592	-0.983585	2.265726
C	6.312495	-1.796170	2.351392
O	6.217002	-3.032705	1.566418
C	7.097787	-2.984060	0.410828
C	7.572552	-1.081036	1.817960
C	7.655260	-1.549267	0.348291
H	8.450498	-1.418027	2.379646
H	4.155750	-1.653426	2.347968
H	5.011944	-0.257855	3.087025
H	6.424682	-2.099980	3.398836
H	7.890084	-3.720908	0.543837
H	7.487240	0.007187	1.901666
H	7.027441	-0.917301	-0.284153
H	8.676079	-1.548734	-0.045369
N	6.340067	-3.417210	-0.783702
C	6.594843	-4.698124	-1.376369
O	7.513759	-5.418165	-0.926446

N	5.809671	-5.092276	-2.421724
C	4.816186	-4.304785	-2.867188
N	4.062081	-4.756503	-3.894088
C	4.536973	-3.021769	-2.292661
C	5.309384	-2.635551	-1.242160
H	4.186712	-5.704333	-4.230030
H	3.234343	-4.250489	-4.181542
H	3.718293	-2.405761	-2.648433
H	5.139019	-1.714206	-0.698558
Na	6.096078	3.720161	0.575727

Nad(GpG), Total Bonding Energy: -8861.39 kcal/mol

H	-0.042565	0.482899	-0.309279
C	0.694299	0.338440	0.491257
O	0.745856	-1.089208	0.849819
C	1.991094	-1.643135	0.342851
C	2.094616	0.692391	-0.014836
C	2.504203	-0.626406	-0.679607
O	2.910225	1.025111	1.163156
H	0.407785	0.908574	1.380067
H	2.703555	-1.761570	1.161174
H	2.100579	1.543800	-0.700147
H	1.953763	-0.729707	-1.622468
H	3.570230	-0.729395	-0.872371
N	1.772673	-2.981072	-0.188063
C	0.945265	-3.380916	-1.230226
N	1.060066	-4.665200	-1.516534
C	2.024703	-5.137873	-0.627779
C	2.614458	-6.432020	-0.483901
O	2.391014	-7.479042	-1.124963
N	3.602146	-6.427432	0.541187
C	3.982109	-5.335280	1.299230
N	4.971956	-5.510753	2.227907
N	3.413639	-4.144348	1.173062
C	2.474745	-4.103155	0.204901
H	0.280483	-2.680572	-1.717880
H	4.126083	-7.296269	0.640936
H	5.550224	-6.337134	2.109968
H	5.521561	-4.655976	2.372761
P	4.554864	1.001579	1.131280
O	5.081514	1.363930	-0.247228

O	5.007427	1.856269	2.302657
O	4.928389	-0.569448	1.352840
C	4.976120	-1.193430	2.670154
C	6.347497	-1.822690	2.907962
O	6.550180	-3.014189	2.076979
C	7.322470	-2.685371	0.897303
C	7.550771	-0.920826	2.573941
C	7.866477	-1.250701	1.092485
H	8.395425	-1.189704	3.216624
H	4.206413	-1.973064	2.695347
H	4.791068	-0.447504	3.452493
H	6.365433	-2.173669	3.946325
H	8.117598	-3.431325	0.832408
H	7.312500	0.138879	2.713459
H	7.360788	-0.543277	0.435778
H	8.938105	-1.223979	0.878138
N	6.517490	-2.870298	-0.326701
C	5.865280	-1.940392	-1.132997
N	5.205181	-2.491978	-2.133062
C	5.413132	-3.858536	-1.980353
C	4.939201	-4.961476	-2.755498
O	4.234721	-4.963084	-3.783804
N	5.385114	-6.194437	-2.200613
C	6.192421	-6.335183	-1.090683
N	6.426258	-7.612312	-0.653922
N	6.663207	-5.306023	-0.400901
C	6.229479	-4.108990	-0.869021
H	5.895126	-0.880955	-0.925154
H	5.043493	-7.030768	-2.669857
H	6.333096	-8.370873	-1.321478
H	7.197240	-7.714143	-0.001494
Na	6.317836	3.298154	0.736926

Nad(GpT), Total Bonding Energy: -8557.97 kcal/mol

H	0.110686	-0.847094	0.333576
C	0.881544	-0.522748	1.048447
O	1.740002	-1.646015	1.414673
C	2.667287	-1.840626	0.326610
C	1.832447	0.468250	0.379697
C	2.754831	-0.481188	-0.418817
O	2.487254	1.222361	1.449599

H	0.417134	-0.141788	1.960918
H	3.613721	-2.169449	0.759379
H	1.319291	1.178440	-0.274940
H	2.376479	-0.560055	-1.439786
H	3.779154	-0.120048	-0.474748
N	2.210449	-2.927870	-0.558057
C	1.218665	-2.873553	-1.533682
N	0.973465	-4.041798	-2.093865
C	1.840363	-4.921557	-1.452512
C	2.029363	-6.327013	-1.630476
O	1.457834	-7.105167	-2.419730
N	3.027445	-6.817110	-0.746366
C	3.729329	-6.067570	0.179343
N	4.622416	-6.734447	0.963705
N	3.546304	-4.762013	0.342133
C	2.607987	-4.250844	-0.488380
H	0.722357	-1.945304	-1.779895
H	3.214824	-7.815771	-0.812231
H	4.908620	-7.666810	0.687557
H	5.348380	-6.169125	1.394226
P	3.964301	1.918236	1.286712
O	4.206649	2.412092	-0.127500
O	4.065742	2.934698	2.411419
O	4.990042	0.663924	1.523553
C	4.921182	-0.022993	2.813497
C	6.032880	-1.053237	2.908126
O	5.734170	-2.171417	2.000670
C	6.906852	-2.436681	1.195158
C	7.444573	-0.539244	2.512282
C	7.645796	-1.097242	1.089263
H	8.194293	-0.954697	3.194041
H	3.942882	-0.511147	2.908273
H	5.045495	0.707683	3.623153
H	6.025643	-1.439267	3.935353
H	7.535391	-3.185019	1.683515
H	7.499030	0.552922	2.543441
H	7.161690	-0.445773	0.355961
H	8.696609	-1.238394	0.818539
N	6.466447	-3.033363	-0.075878
C	6.705923	-4.390479	-0.306046
O	7.284616	-5.138515	0.493509

N	6.239927	-4.847514	-1.527326
C	5.501742	-4.139589	-2.490904
O	5.138929	-4.708227	-3.533815
C	5.231618	-2.750471	-2.143772
C	4.418988	-1.912954	-3.096498
C	5.727511	-2.275439	-0.971512
H	6.436006	-5.825846	-1.729440
H	4.868226	-1.925566	-4.097804
H	4.354631	-0.877756	-2.748525
H	3.402609	-2.318996	-3.194174
H	5.555282	-1.253936	-0.655482
Na	4.597350	4.725316	0.740501

Nad(TpA), Total Bonding Energy: -8393.06 kcal/mol

H	0.503260	-0.322911	-0.199098
C	1.035708	-0.194451	0.754504
O	1.270572	-1.493127	1.385153
C	2.515698	-2.032872	0.873893
C	2.428310	0.367891	0.494797
C	3.203682	-0.895688	0.084880
O	2.918995	0.939552	1.751534
H	0.438726	0.410256	1.442220
H	3.121492	-2.365603	1.714232
H	2.440455	1.140600	-0.278445
H	3.114467	-1.036287	-0.995113
H	4.259236	-0.843598	0.344531
N	2.247136	-3.248739	0.064213
C	2.472151	-4.505768	0.644815
O	2.945966	-4.674889	1.772085
N	2.108913	-5.571776	-0.166926
C	1.451500	-5.533938	-1.407661
O	1.128228	-6.591860	-1.973334
C	1.201076	-4.192385	-1.915103
C	0.505921	-4.031277	-3.242280
C	1.596929	-3.136375	-1.157143
H	2.276601	-6.493277	0.231224
H	-0.490153	-4.493557	-3.217338
H	1.074834	-4.535673	-4.034756
H	0.400318	-2.970959	-3.497158
H	1.409878	-2.119961	-1.485014
P	4.356548	1.736382	1.784320

O	4.584732	2.515117	0.501602
O	4.366847	2.509375	3.092113
O	5.472412	0.539441	1.796430
C	5.492620	-0.376467	2.935973
C	6.393063	-1.562594	2.626425
O	5.725214	-2.429689	1.646400
C	6.675939	-2.752660	0.602768
C	7.776521	-1.202546	2.014759
C	7.604118	-1.537675	0.519721
H	8.552869	-1.834796	2.458391
H	4.475216	-0.731048	3.144452
H	5.878904	0.152804	3.815976
H	6.509757	-2.127194	3.560606
H	7.229034	-3.658707	0.879116
H	8.033004	-0.152167	2.181039
H	7.094717	-0.715262	0.008048
H	8.542782	-1.769719	0.007980
N	5.951199	-3.079689	-0.617456
C	5.541304	-2.236415	-1.638134
N	4.894680	-2.855289	-2.610452
C	4.878385	-4.189803	-2.212851
C	4.377436	-5.363499	-2.824767
N	3.753029	-5.346995	-4.026930
N	4.559629	-6.550471	-2.189153
C	5.197682	-6.553415	-1.000024
N	5.711235	-5.511339	-0.319966
C	5.527023	-4.351114	-0.980019
H	5.752515	-1.177647	-1.607865
H	3.280102	-6.183061	-4.348450
H	3.503536	-4.460155	-4.445224
H	5.312087	-7.529439	-0.532718
Na	4.752095	4.639678	1.822169

Nad(TpC), Total Bonding Energy: -7994.04 kcal/mol

H	6.577118	-14.654767	-5.304977
C	7.327915	-14.529539	-4.512549
O	7.611789	-13.104476	-4.313660
C	6.803727	-12.607725	-3.216193
C	6.762821	-15.015186	-3.181602
C	5.940231	-13.795524	-2.744233
O	7.893911	-15.291783	-2.294109

H	8.261589	-15.024512	-4.793192
H	7.463488	-12.247292	-2.425634
H	6.153642	-15.917650	-3.278142
H	4.978996	-13.825548	-3.266030
H	5.747301	-13.777434	-1.674109
N	6.020203	-11.433648	-3.657206
C	6.361509	-10.164269	-3.166332
O	7.329702	-9.942050	-2.437250
N	5.491426	-9.158904	-3.567766
C	4.402401	-9.253814	-4.447035
O	3.727635	-8.244063	-4.712820
C	4.176704	-10.587634	-4.989585
C	3.066715	-10.793374	-5.989143
C	4.991771	-11.592942	-4.574423
H	5.702701	-8.233721	-3.198758
H	3.223011	-10.162494	-6.874705
H	2.098069	-10.506965	-5.557353
H	3.020337	-11.841998	-6.303051
H	4.874090	-12.600129	-4.959171
P	7.657504	-15.586308	-0.691305
O	6.248559	-16.082711	-0.429637
O	8.811224	-16.468949	-0.248700
O	7.763634	-14.097879	-0.014346
C	9.067763	-13.431773	-0.005792
C	9.089179	-12.306810	1.021730
O	8.271250	-11.183304	0.558183
C	7.198444	-10.920175	1.496458
C	8.549644	-12.675321	2.424983
C	7.094687	-12.166092	2.395686
H	9.126697	-12.144803	3.190149
H	9.266930	-13.026946	-1.006438
H	9.845847	-14.160125	0.254642
H	10.128514	-11.957964	1.072883
H	7.426927	-10.023699	2.075516
H	8.610910	-13.751766	2.614356
H	6.442091	-12.915149	1.938812
H	6.705562	-11.906353	3.385099
N	5.976622	-10.609590	0.725553
C	5.429564	-9.287343	0.711285
O	5.954667	-8.389983	1.406805
N	4.331472	-9.052809	-0.068994

C	3.800438	-10.034930	-0.815281
N	2.715581	-9.746442	-1.565982
C	4.352836	-11.358605	-0.848705
C	5.444250	-11.587727	-0.071251
H	2.327932	-8.810714	-1.564649
H	2.278647	-10.454614	-2.140349
H	3.932333	-12.135208	-1.477007
H	5.964154	-12.537789	-0.047588
Na	7.169772	-18.185014	0.564910

Nad(TpG), Total Bonding Energy: -8558.23 kcal/mol

H	5.092019	-13.842928	-4.481985
C	6.139737	-13.999128	-4.189130
O	6.938310	-12.822295	-4.551908
C	7.002569	-11.930338	-3.426608
C	6.236952	-14.113883	-2.669721
C	6.309692	-12.642613	-2.245466
O	7.455833	-14.847795	-2.323729
H	6.553664	-14.860645	-4.719275
H	8.044114	-11.677127	-3.231557
H	5.373476	-14.630540	-2.241054
H	5.288790	-12.264952	-2.134344
H	6.839258	-12.484916	-1.307083
N	6.319160	-10.639304	-3.744823
C	6.934615	-9.436815	-3.396722
O	8.094058	-9.340629	-2.980677
N	6.108998	-8.325916	-3.543207
C	4.770029	-8.294644	-3.954558
O	4.135023	-7.221359	-3.919297
C	4.227040	-9.579536	-4.364326
C	2.807406	-9.648920	-4.863500
C	5.018481	-10.676308	-4.221156
H	6.527243	-7.437535	-3.274571
H	2.656118	-8.949282	-5.695748
H	2.106843	-9.373554	-4.063602
H	2.567719	-10.663966	-5.198548
H	4.661331	-11.667552	-4.479332
P	7.587216	-15.498385	-0.816555
O	6.281515	-16.131164	-0.373186
O	8.822206	-16.381782	-0.862145
O	7.812856	-14.206232	0.159401

C	9.021998	-13.405673	-0.008064
C	8.971461	-12.199469	0.922578
O	7.889279	-11.294792	0.521872
C	6.820781	-11.326830	1.500698
C	8.708768	-12.506478	2.411764
C	7.172656	-12.411084	2.532975
H	9.190957	-11.740309	3.028949
H	9.089584	-13.061055	-1.048406
H	9.902700	-14.016578	0.228059
H	9.913550	-11.655336	0.782692
H	6.757327	-10.333117	1.953778
H	9.089008	-13.490878	2.703750
H	6.723850	-13.366772	2.259619
H	6.834211	-12.122873	3.532167
N	5.535660	-11.526858	0.814866
C	4.798635	-12.689042	0.607329
N	3.708444	-12.489450	-0.109221
C	3.731554	-11.130849	-0.412907
C	2.836831	-10.336514	-1.197974
O	1.787256	-10.671787	-1.777534
N	3.296761	-8.992746	-1.279725
C	4.432815	-8.485047	-0.677083
N	4.672890	-7.152128	-0.845689
N	5.252475	-9.227517	0.058235
C	4.860948	-10.521122	0.149925
H	5.127074	-13.645565	0.986491
H	2.719582	-8.363110	-1.834184
H	4.305487	-6.709580	-1.685155
H	5.592535	-6.837513	-0.554014
Na	7.438647	-18.333168	-0.099011

Nad(TpT), Total Bonding Energy: -8252.98 kcal/mol

H	6.600541	-14.506046	-5.617104
C	7.492229	-14.403524	-4.983432
O	7.813979	-12.982350	-4.809861
C	7.317780	-12.537919	-3.518645
C	7.194309	-14.931200	-3.582713
C	6.518647	-13.716985	-2.934408
O	8.472443	-15.244176	-2.936215
H	8.349373	-14.893576	-5.453991
H	8.158823	-12.273667	-2.878973

H	6.564195	-15.824703	-3.589094
H	5.469917	-13.682615	-3.247354
H	6.576193	-13.724718	-1.848385
N	6.530714	-11.300090	-3.680744
C	7.048213	-10.089599	-3.195981
O	8.168378	-9.959228	-2.699132
N	6.162800	-9.024211	-3.312654
C	4.926529	-8.992647	-3.978974
O	4.270882	-7.936958	-4.015890
C	4.531484	-10.258526	-4.583001
C	3.252622	-10.326728	-5.377274
C	5.342209	-11.332987	-4.395833
H	6.498718	-8.140580	-2.934070
H	3.283378	-9.621344	-6.218745
H	2.399242	-10.053609	-4.744640
H	3.097374	-11.338514	-5.768598
H	5.095656	-12.300031	-4.820857
P	8.489682	-15.966602	-1.457553
O	7.366264	-16.978592	-1.330032
O	9.913104	-16.458917	-1.260519
O	8.133176	-14.757263	-0.414242
C	9.073160	-13.646509	-0.285628
C	8.423447	-12.506271	0.479083
O	7.379632	-11.917047	-0.349786
C	6.432012	-11.323140	0.555985
C	7.745692	-12.880067	1.837371
C	6.299041	-12.338026	1.705569
H	8.277878	-12.398683	2.663385
H	9.372358	-13.296680	-1.281552
H	9.963287	-13.985911	0.257352
H	9.203636	-11.748419	0.644134
H	6.800940	-10.360362	0.921146
H	7.750767	-13.962181	1.996040
H	5.610322	-13.137744	1.414399
H	5.927418	-11.865588	2.619913
N	5.199646	-11.047557	-0.198111
C	4.777972	-9.725343	-0.382001
O	5.367005	-8.737343	0.070034
N	3.612953	-9.609298	-1.126369
C	2.852927	-10.632972	-1.719283
O	1.807000	-10.354082	-2.327918

C	3.403123	-11.969964	-1.544214
C	2.721396	-13.135730	-2.212426
C	4.521394	-12.106096	-0.786169
H	3.279350	-8.657087	-1.264638
H	1.679017	-13.224008	-1.879255
H	3.245516	-14.071488	-1.988977
H	2.698293	-12.990782	-3.301691
H	4.965651	-13.076424	-0.606064
Na	9.044523	-18.774735	-0.844816
